# Multifaceted interventions for supporting community participation among adults with disabilities: A systematic review

**DOI:** 10.1002/cl2.1092

**Published:** 2020-06-23

**Authors:** Judith M. S. Gross, Amalia Monroe‐Gulick, Chad Nye, Debbie Davidson‐Gibbs, Devin Dedrick

**Affiliations:** ^1^ Center on Community Living and Careers, Indiana Institute on Disability and Community Indiana University Bloomington Indiana; ^2^ University of Kansas Lawrence Kansas; ^3^ American Institutes for Research Lakeland Florida; ^4^ American Institutes for Research Washington District of Columbia; ^5^ American Institutes for Research Austin Texas

## 1 PLAIN LANGUAGE SUMMARY

### 1.1 Multifaceted interventions show limited impact on community participation among adults with disabilities

Multifaceted interventions combine different intervention components, such as social skills training and work experience, to improve community participation outcomes for people with disabilities. The evidence shows limited support for the approach. More and better evidence is needed.

### 1.2 What is this review about?

Multifaceted interventions are interventions which target two or more individual or environmental characteristics in different domains. For example, many factors affect the outcome of integrated, competitive employment in the community for people with disabilities. Among those factors, there are points of intervention related to the individual (e.g., work experience, social skills, level of support needs, education/training), the employer or workplace (e.g., disability awareness, provision of accommodations, accessibility), and the community (e.g., access to transport, proximity to workplace).

This review examines multifaceted interventions that measure outcomes relevant to community participation for adults with disabilities.

**What is the aim of this review?**

This Campbell systematic review and meta‐analysis examines the impact of multifaceted interventions on community participation outcomes for adults with disabilities, and aims to find effective components of the interventions. The review summarizes the findings from 15 reports of multifaceted interventions in five countries.

### 1.3 What studies are included?

Included studies employ at least two interventions designed to address two or more participant characteristics (e.g., skill enhancement, behavior/attitude change) and/or environmental characteristics (e.g., participant interactions with people, places, or things) resulting in outcomes that provide direct access to the community (e.g., competitive employment, adult learning, housing) or are a dimension of community participation (e.g., self‐determination, quality of life, social networking).

A total of 15 studies using a multifaceted intervention were included in this review. Of these, nine were randomized and six were quasiexperimental. Study participants were adults, 18 years or older, with a disability, who had exited secondary school services. Participants identified as having the following disabilities: intellectual disability, mental illness, traumatic brain injury (TBI), aging‐related disabilities (e.g., dementia, Alzheimer's, reduction in activities of daily living), or combinations of two or more classifications.

### 1.4 What are the main findings of this review?

Individual studies of multifaceted interventions focus on increasing community participation of adults with disabilities. These studies show evidence of positive effects for some outcomes (employment, quality of life, and adult learning). However, there are no significant effects on other outcomes (activities of daily living, mental health, autonomy, independent living, social skills, community activities, and housing).

The evidence supporting multifaceted interventions is hampered by (a) lack of design quality in the studies and (b) the small number of studies represented in each multifaceted intervention and the associated outcome.

### 1.5 What do the findings of this review mean?

#### 1.5.1 Implications for research

Limited support for the effectiveness of multifaceted over single‐faceted interventions suggests the need for more substantial research to determine effectiveness broadly as well as specifically in relation to community participation of adults with disabilities. Future research should narrow the focus to more specific outcomes for targeted groups of adults with similar disabilities, which may yield greater insight into the potential effectiveness of multifaceted interventions.

Disability populations that are most frequently the target of multifaceted interventions tend to need greater support for executive functioning. Further, the multifaceted interventions for these populations tend to include cognitive coaching as one of the facets of the intervention. Therefore, more research on multifaceted interventions targeting these populations may provide greater insight into the effectiveness of multifaceted interventions across specific outcomes of interest (e.g., competitive employment).

#### 1.5.2 Implications for policy

The studies included in this systematic review did not directly address policy. However, employment and community living for people with disabilities are important policy areas. As such, continued research is needed to more effectively inform and guide future policies that will support community participation for adults with disabilities. More research evidence from high quality studies is needed before policy implications can be formed.

#### 1.5.3 How up‐to‐date is this review?

The review authors searched for studies up to the end of 2016.

## 2 EXECUTIVE SUMMARY/ABSTRACT

### 2.1 Background

Intervention research typically assesses the impact of one intervention intended to produce a desired change or outcome. Often, however, the context of the setting or needs of the participants reveal multiple points of intervention that could be addressed to achieve the desired outcome. This possibility is especially likely when the research takes place in the real‐life context of community participation among people with disabilities. For example, many people with disabilities have difficulty attaining competitive employment (an outcome that provides the person with a disability with direct access to his/her community). Previous research identified numerous barriers to employment, including but not limited to poor social skills, lack of work experience, lack of transportation, discriminatory hiring practices, and lack of needed on‐the‐job supports. Each of these barriers presents a possible point for intervention.

### 2.2 Objectives

In this study, we examine the effects of multifaceted interventions that address two or more participant characteristics (e.g., social skills, work experience) and/or environmental characteristics (e.g., transportation, hiring practices, on‐the‐job supports) resulting in outcomes that provide direct access to the community (e.g., competitive employment) or are a dimension of community participation (e.g., quality of life, social networking) for adults with disabilities.

### 2.3 Search methods

After reviewing 15 databases and one publisher journal package, we selected three databases for initial peer‐reviewed literature electronic searching: PubMed, Web of Science, and PsycInfo. We selected these databases based on guidelines in the Cochrane Handbook on database selection, including the necessity of searching of PubMed, and the inclusion of both a general bibliographic and a subject‐specific database (Higgins & Green, 2011). The search strategies are informed by previous systematic reviews (Peterson‐Besse et al., 2014; White, Monroe‐Gulick, & O'Brien, 2013). The goal is to ensure that all types of disabilities are included in the search results, but also exclude irrelevant results. In addition, we considered that each database has a specific search strategy because of the different controlled vocabulary and search mechanisms, and we used the vocabulary and search strategies most effective for each database to improve and narrow the search results to more accurately represent our research questions.

We also searched two additional databases, Dissertations and Theses Abstracts and PolicyFile, to find potentially relevant “grey” literature. These two databases were selected because they offer a variety of literature as well as controlled search environments. PolicyFile's sources include think tanks, universities, publishers, and nonprofits, both domestic and international. Dissertation and Theses Abstracts provides potentially more current and comprehensive research than the peer‐reviewed literature.

### 2.4 Selection criteria

This review includes studies that employed multifaceted interventions to improve community participation outcomes for adults with disabilities who had exited the school system. Multifaceted interventions seek to change/impact two or more personal characteristics or environmental factors that are in different domains (e.g., employability skills and transportation skills). Community participation outcomes include those outcomes that are chosen by/desired by the individual with a disability, occur in the community (i.e., integrated with people without disabilities), and are reflective of (a) direct access to or participation in the community or (b) dimensions of community participation (i.e., outcomes for which there is a research base linked to community participation). Selected studies include quantitative randomized controlled trial (RCT) and quasiexperimental designs (QEDs).

### 2.5 Data collection and analysis

At least two review authors collaboratively determined inclusion and exclusion decisions through screening titles, abstracts, and full‐text articles of the search results. A total of 37 studies were identified as meeting inclusionary criteria. However, only 15 of these were assessed to be of sufficient methodological quality and with data collection methods appropriate to be included in the final analysis. We used an adapted version of the National Technical Assistance Center on Transition (NTACT) Quality Indicator Checklists for Group Experimental studies (see, http://www.transitionta.org/effectivepractices) to assess the methodological quality of the selected articles. Two of the studies met the criteria of “high quality” as defined by the items on the NTACT checklist. The remaining 13 studies were in the category of “acceptable quality.”

At least two review authors independently extracted data for all eligible studies and then reviewed the data for accurate entry. When two or more study samples provided sufficient information to permit effect size calculations, we conducted random‐effects meta‐analyses to synthesize effects across studies.

### 2.6 Results

Our search located 15 eligible studies measuring outcomes related to community participation, including those with direct access to the community (e.g., employment, adult learning, recreation, housing) and those outcomes associated with or dimensions of community participation (e.g., physical health, self‐determination, social networking, quality of life).

A total of 74 effect sizes were calculated across the 15 studies, representing 3,296 treatment participants and 3,782 control or comparison participants. All included studies employed a multifaceted intervention with participants with disabilities. A meta‐analysis of the impact on the outcomes at the study level was possible for the following community participation outcomes: employment, quality of life, mental health, adult learning, activities of daily living, autonomy, independent living, social skills, community activities, and housing. Positive treatment effects were found for three of these outcomes: employment, quality of life, and adult learning. Results of the aggregation of studies based on the method of intervention for each of the outcome areas yielded a nonsignificant overall effect size.

The evidence supporting multifaceted interventions is hampered by (a) the lack of design quality reflected in the studies and (b) the small number of studies represented in each multifaceted study and the associated outcome. Further research should focus on the classification of the interventions.

### 2.7 Authors' conclusions

This systematic review on multifaceted interventions targeting community participation outcomes suggests limited effects of using multifaceted interventions, and that more research is needed to determine whether such strategies are worthwhile.

## 3 BACKGROUND

### 3.1 The problem, condition, or issue

#### 3.1.1 History of legislation

Numerous U.S. laws have been passed to increase protections and opportunities for people with disabilities to live more independently in the community. Section 504 of the Rehabilitation Act of 1973 was created to increase opportunity for physical and programmatic access in institutions (e.g., universities) that received federal monies. Title VII of the Rehabilitation Act established Centers for Independent Living, and mandated four core services: information and referral, advocacy, peer counseling, and independent living skills training; a fifth core service, transition, was added in the latest version of the Workforce Innovation and Opportunity Act of 2014 (Administration for Community Living [ACL], 2016). The Fair Housing Amendments Act of 1988 allowed tenants to make reasonable modifications to their apartments at their own expense; the Americans with Disabilities Act of 1990 was designed to protect the civil rights of citizens with disabilities; the *Olmstead v. L.C*. Supreme Court decision (1999) asserted people's right to live in the least restrictive environments.

Despite these enacted laws, many people with disabilities lack personal knowledge, skills, resources, and/or enabling environments that would allow them to take advantage of these rights and resources and to live more independently in the community. Thus, multifaceted interventions for people with disabilities that simultaneously address personal growth, such as job skills, while also building knowledge of one's rights, such as how to request reasonable accommodations, have a unique potential to truly result in increased participation for people with disabilities. Moreover, resulting increased participation in the community by people with disabilities also increases awareness of disability issues among the nondisabled population and possible support for further improvements in policies to facilitate even greater participation.

#### 3.1.2 Rehabilitation research and training center (RRTC)

The University of Kansas, in partnership with the University of Montana, operates a RRTC on Promoting Interventions for Community Living (RRTC/PICL). The purpose of the Center is to examine the effectiveness of interventions to support greater community participation for individuals with physical and co‐occurring multiple disabilities and to promote the dissemination and utilization of effective interventions. The RRTC/PICL is charged with developing and promoting interventions that support individuals with disabilities and positively impact community participation outcomes for such individuals. One of the goals of RRTC/PICL is to identify key components of effective evidence‐based, multifaceted interventions that support individuals with disabilities in the community through a systematic review of the literature.

### 3.2 The intervention

The systematic review focused on identifying studies with multifaceted interventions that promote community living among people with disabilities. Multifaceted interventions that measured outcomes relevant to community participation were examined. A community participation intervention is defined as something that is done to, with, or for the person with a disability or the environment in which they interact or want to interact with to achieve community participation outcomes. We rely heavily on research conducted by our research team member Bryce Ward, Ph.D., the Associate Director at the Bureau of Business and Economic Research and Director of the Bureau's Health Care Research Program at the University of Montana.

#### 3.2.1 Definitions

Definitions related to the intervention(s) that were part of the systematic review of literature are as follows.

- *Multifaceted interventions*—interventions that address two or more individual (changing something about the person—enhancing skills/knowledge, changing behavior, changing perceptions/attitudes) or environmental characteristics (changing something about the people, places, or things in the environments in which the person interacts) in different domains (e.g., social skills, financial management, physical health, mental health, employment, adult learning, health care).
- *Disability*—physical or cognitive limitations, including multiple disabilities (Note: In an effort to narrow the targeted population, we focused on disability types relevant to the RRTC/PICL project, for which this systematic review was conducted as a foundational activity to inform the grant's research. Therefore, we excluded several disabilities outside of the Center's targeted stakeholders. See Section 5.1.2, for a complete list of the exclusions).
- *Community*‐based settings—settings that are integrated with people without disabilities in an environment where people without disabilities typically work, live, and recreate.
- *Community participation outcomes*—are chosen by/desired by the individual with a disability, occur in the community (i.e., integrated with people without disabilities), and are reflective of (a) direct access to or participation in the community or (b) dimensions of community participation (i.e., outcomes for which there is a research base linking them to community participation). Below we have provided examples of what might be included in each broad outcome category. However, we do not want to limit our community participation outcomes by strictly defining each term below.
- *Direct access to or participation in the community*
  - Integrated competitive employment
  - Adult learning
    ▪ Education
    ▪ Training
  - Housing
    ▪ Place
    ▪ Housemates
    ▪ Usability
  - Civic involvement
    ▪ Voting
    ▪ Advocacy
    ▪ Committees/leadership
    ▪ Volunteer work
  - Recreation
    ▪ Sports
    ▪ Arts
    ▪ Music
    ▪ Community events (e.g., art in the park, parades, block parties)
  - Navigating the community (e.g., transportation)
- *Dimension of community participation*
  - Increased self‐determination
    ▪ Autonomy
    ▪ Self‐advocacy
  - Improved health
    ▪ Physical
    ▪ Mental
  - Improved quality of life
  - Increased family support/activities in the home
    ▪ Caregiving
    ▪ Supporting children/parenting
    ▪ Household chores/care
  - Social networking
    ▪ Friendships
    ▪ Relationships (e.g., boy/girlfriend, spouse)
    ▪ Church/religious activities.

### 3.3 Multifaceted interventions described

We define *multifaceted interventions* as interventions that target two or more individual or environmental characteristics in different domains. To “target individual characteristics” can be understood as seeking to change something about the person (e.g., enhancing skills/knowledge, changing behavior, changing perceptions/attitudes). For example, an intervention may seek to change someone's behavior with regard to how they interact with their employer (domain of social skills or soft skills). To “target environmental characteristics” can be understood as seeking to change something about the people, places, or things in the environments in which the person interacts. For example, an intervention may seek to change people in the environment (e.g., train employers or coworkers on how to provide natural supports for their coworker with a disability), change the physical work place (e.g., increasing spacing between furniture), or change things used in the workspace (e.g., use of visual supports for following task directions). Two interventions (e.g., soft skills training for the employee and use of visual supports for following task directions) could be combined to create a multifaceted intervention targeting increasing duration of employment for people with intellectual disabilities.

Another example of a multifaceted intervention is when the goal to increase community participation for adults with disabilities is addressed by employing two different interventions, one to address increasing social engagement (e.g., participation in community groups and events) with people in the community and another to provide transportation training to support the person in accessing their community. This is a multifaceted intervention because it addresses two different domains (social engagement and transportation) to impact the outcome of community participation; in this case, both interventions are focused on changing something about the person (behavior and knowledge).

A nonexample of a multifaceted intervention would be if the goal to increase community participation for adults with disabilities used only one intervention targeting social engagement or multiple interventions targeting social engagement. This is not a multifaceted intervention by our definition because there is only one domain (social engagement) that the interventions being implemented are focused on (a multifaceted intervention aims to change at least two or more individual or environmental characteristics in different domains).

### 3.4 How the intervention might work

**Figure 1:**
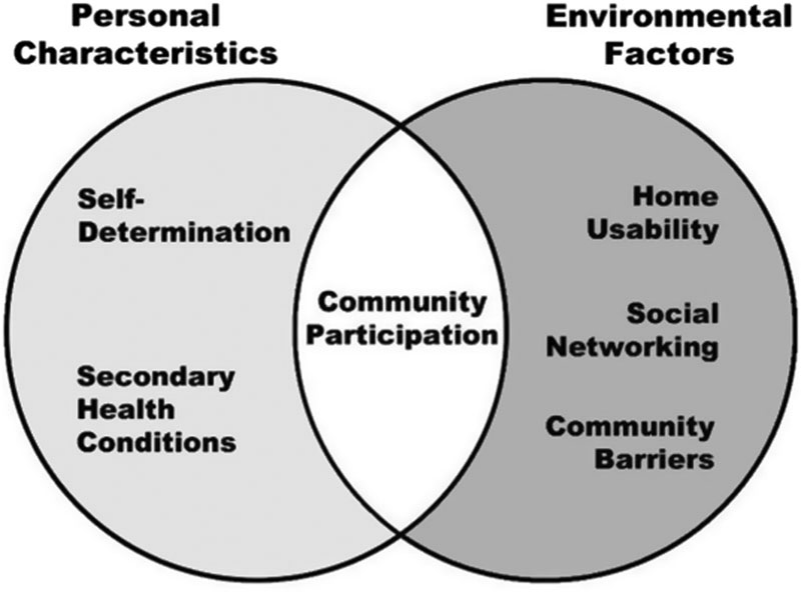
Adapted person‐environment fit model with examples of personal characteristics and environmental factors

The theoretical framework that guided our work on this systematic review of literature related to community participation interventions is the concept of person‐environment fit. The person‐environment diagram (see Figure 1), adapted from Tang et al. (2011), depicts the interaction of personal and environmental factors.

This theoretical framework meshes with the Institute of Medicine's model of disability as a dynamic process that results from characteristics and interactions of the individual with his/her environment (Brandt & Pope, 1997; Field & Jette, 2007; McDermott & Turk, 2011; Nagi, 1991). “Disability” itself “depends on an interaction between the individual and the physical and social environment” (Rehabilitation Act (Sec. 2(a)(3)). Person‐environment fit theory has been applied in numerous areas, including employee attitudes and behaviors (Duffy, Autin, & Bott, 2015), functioning among older adults in long‐term care settings (Rantakokko, Törmäkangas, Rantanen, Haak, & Iwarsson, 2013), and transition of youth with disabilities to adulthood (Stewart et al., 2014).

In addition to our theoretical framework, we subscribe to the basic values of Independent Living Philosophy (DeJong, 1978; Pfeiffer, 2002; White, Lloyd Simpson, Gonda, Ravesloot, & Coble, 2010). These values are consistent with the basic guidelines for quality Home and Community‐Based Services (HCBS) related to the provision of Medicaid services to people with disabilities in the community (National Quality Forum, 2015). These values also provide the ethical and social underpinnings for intervention research that go beyond empirically derived best practices. They include, among others, services provided to maximize integration and inclusion in the community; enabling self‐direction, choice, and control; adherence to human and legal rights to personal freedom, privacy, and risk balanced with safety and support; and delivering and managing services that are coordinated, dependable, and comprehensive (National Quality Forum, 2015).

Considering this theoretical and value‐based orientation, we reviewed studies that take into account various personal characteristics as well as environmental factors. Personal characteristics are often the target of interventions seeking to change something about the person (how they think, what they know, how they act). Environmental factors may be the target of interventions seeking to increase access and opportunity for people with disabilities. We reviewed studies of interventions that address two or more of these characteristics and factors across different domains and examined the effects of the intervention on community participation outcomes for a diversity of people with disabilities.

### 3.5 Why it is important to do the review

#### 3.5.1 Existing research on disabilities and community participation

Studies of the impact of single‐faceted interventions on community participation of adults with disabilities are numerous. An example of a systematic review of a single intervention is a study of the efficacy of using service dogs for people with physical disabilities (Winkle, Crowe, & Hendrix, 2012). Other reports examining the impact of a single intervention on community participation for adults with disabilities include a study of transportation vouchers (Samuel, Lacey, Giertz, Hobden, & LeRoy, 2013), an exercise intervention (Dean et al., 2012), and a cognitive intervention to reduce fear of falling and associated avoidance of activity among older adults with disabilities (Haastregt et al., 2007). However, our theoretical orientation is on reviewing the effectiveness of multifaceted interventions, which are defined as interventions that address two or more aspects for change (Eldh & Wallin, 2015). People with complex and multiple support needs are likely to benefit from more comprehensive, multifaceted interventions that target multiple aspects for change. Our hypothesis is that multifaceted interventions will be more effective, especially when confronted with multiple, complex, and interacting factors affecting outcomes of a complex phenomenon such as community participation by people with a wide range of disabilities.

Very few systematic reviews of multifaceted interventions related to disability issues are in the literature. Most reviews on the topic of disability and community relate to descriptive factors that serve as barriers or facilitators to participation (e.g., White, Gonda, Peterson, & Drum, 2011). In searching The Cochrane Collaborative, The Campbell Collaboration, and NARIC databases, we found no systematic reviews of multifaceted interventions intended to enhance community participation by adults with disabilities of any type. Williams et al. (2007) conducted a systematic review of multifaceted interventions to improve depression care and found strong evidence for the effectiveness of care management for depression; however, we did not use the same definition of “multifaceted intervention” as they did. Williams et al. described “multifaceted interventions” through their inclusionary criteria, indicating that an intervention targeting depression would be included in the review if it included usual care and incorporated “at least one patient‐directed element” of the Chronic Care Model, which is a healthcare specific model. Therefore, this definition of a “multifaceted intervention” would not have been applicable to our search for multifaceted interventions to promote the community living and participation of adults with disabilities.

#### 3.5.2 Research questions and logic model

Reviews of multifaceted interventions on disability or other topics are rarely conducted. However, a systematic review was appropriate in this case because we have clear questions and definitions to guide the search for relevant studies. Specifically, the research questions for this systematic review are:

(1) What are the reported community participation outcomes of multifaceted interventions targeted on adults with disabilities?
(2) What are the identified components of effective multifaceted interventions?

**Figure 2:**
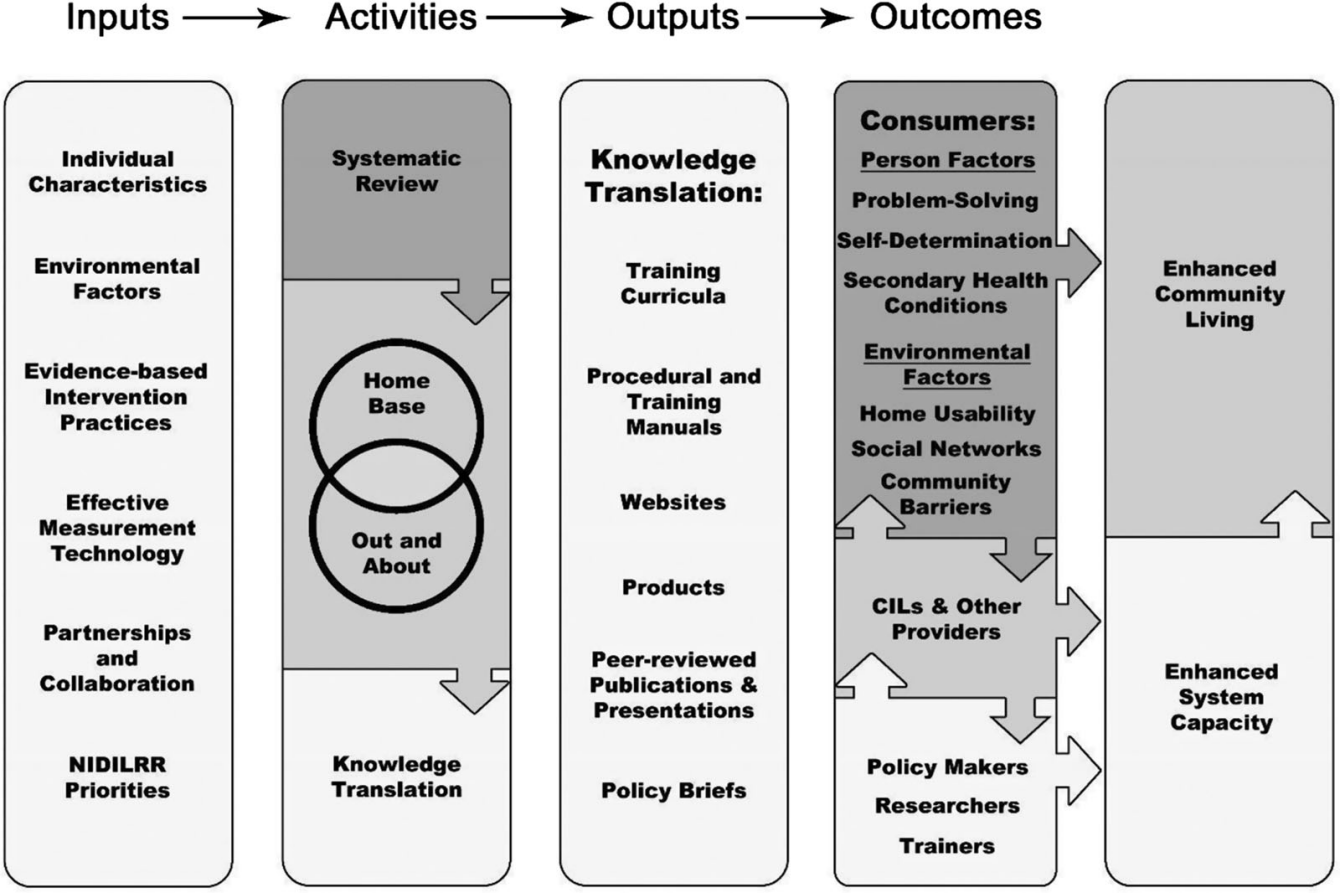
RRTC/PICL logic model. PICL, Promoting Interventions for Community Living; RRTC, Rehabilitation research and training center

Review findings will be used to enhance the intervention research of the previously mentioned RRTC/PICL. The figure below demonstrates the role the systematic review will play in future Center activities (Figure 2).

We seek to identify components of multifaceted interventions that are effective in facilitating community participation for adults with disabilities, particularly those with severe limitations (e.g., cognition, mobility) that impede their participation in the community. The results of this systematic literature review are intended to inform policy makers in their practical decisions about social and behavioral interventions and public policy regarding funding and services to provide such interventions. In addition, the results have the potential to help health and social work practitioners in the field who work directly with people with disabilities to understand and apply the information regarding multifaceted interventions in their daily work.

## 4 OBJECTIVES

### 4.1 The problem, condition, or issue

The purpose of this systematic literature review was to synthesize the research on the effects of multifaceted interventions provided in community settings for the purpose of promoting community participation outcomes of adults with disabilities (18+ years old, no longer receiving secondary education services) in studies using RCTs and quasiexperimental quantitative research design methods.

In conducting this meta‐analysis, we were able to identify the current status of research on multifaceted interventions for community participation outcomes and point to gaps in the literature on such interventions and features that are effective in enhancing community participation.

## 5 METHODS

### 5.1 Criteria for considering studies for this review

#### 5.1.1 Types of studies

The types of intervention studies included are:

- RCTs
- Quasiexperimental

The team ensured that the research of selected studies is of high quality by using an adapted version of the NTACT Quality Indicator Checklists for Group Experimental studies (see, http://www.transitionta.org/effectivepractices) to assess the methodological quality of the studies to be included in the meta‐analysis. Studies that fell in the category of “weak” designs as described on the checklist were not reviewed.

Types of intervention studies that were not included in the review:

- Case studies
- Single subject
- Single group—pre/post
- Qualitative

Because case studies, and single subject designs examine instances of low frequency, such study designs have been excluded. When appropriately designed and executed, case studies and single subject designs can demonstrate strong internal validity. External validity is a more complicated issue for case studies and single subject designs because they require repeated measurements and replication across participants, settings, and researchers to establish an adequate level of external validity (Horner et al., 2005). The purpose of this exclusion is to improve external validity and more closely align with the population our research questions seek explore.

#### 5.1.2 Inclusionary and exclusionary criteria

A priori criteria were developed for the inclusion of studies in this review. Inclusionary criteria were as follows:

- Published 2000–2016
- Original research involving testing an intervention with the following characteristics:
  - Intervention has a clearly defined disability sample
  - Participants are 18+ years of age and exited from secondary education
  - Intervention is applied in a community‐based setting
  - Applied intervention is multifaceted
  - Outcomes included one or more measures of community participation
- English only

The present review seeks to explore populations and interventions that most closely reflect the current literature. For this reason, we only included studies between the years 2000 and 2016. This is to ensure that studies with outdated interventions or dissimilar populations are not included in the review.

We specifically included English‐only studies because the concepts and terminology we are using vary from culture to culture. We struggled to clearly define terms in English for our work because concepts like “community‐based” and “community participation” differ from culture to culture. Although our research group possesses broad and varied skillsets, often it was difficult to come to agreement among the research team as not all members had the same background and historical knowledge of disability culture in the United States. Considering these challenges, we limited the review to English language studies, which helped to ensure reliability in the application of the inclusionary criteria.

Articles were excluded based on the following criteria:

- Articles that were a literature review or compilation of studies
- Intervention studies applied in segregated environments, where the only people without disabilities are those in a service or leadership capacity serving/supporting the adults with disabilities.

We further applied exclusionary criteria to the inclusionary requirement of a focus on people with disabilities in order to narrow the scope of articles and ensure that the review addressed the populations most likely to be served by our community living and participation research center. Thus, we excluded studies with interventions that were primarily medical in nature for target populations categorized as having the following types of disabilities: obesity, asthma, eating disorders, hoarding, type 2 diabetes, substance abuse/addiction, stroke, cancer, human immunodeficiency virus infection/acquired immune deficiency syndrome (HIV/AIDS), phobias, and other medical issues. Articles featuring these populations often were excluded due to the focus of the intervention (outcome was not related to community participation).

#### 5.1.3 Types of participants

The intervention studies included in the review were required to target persons with a disability/ies for at least a portion of the target sample and report outcomes for the disability sample. Samples in selected articles included adults:

- 18 years of age or older,
- With one or more disabilities, and
- Who had exited the secondary education/high school setting and services.

Study samples did not include individuals under 18 years old or who were still participating in a transition program (typically 18–22 years old) in secondary special education.

#### 5.1.4 Types of interventions

The intervention in each study met the following criteria:

- Intervention measures one or more community participation outcomes targeted for change
- Intervention targets persons with a disability/ies for at least a portion of the sample, with outcomes reported on the disability sample
- Intervention is multifaceted, that is, seeks to change/impact two or more personal characteristics or environmental factors that are in different domains (e.g., transportation training skills and employability skills)

#### 5.1.5 Types of outcome measures

##### Primary outcomes

Community participation outcomes are chosen by/desired by the individual with a disability, occur in the community (i.e., integrated with people without disabilities), and are reflective of (a) direct access to or participation in the community or (b) dimensions of community participation (i.e., outcomes for which there is a research base linking them to community participation). The following are primary outcomes with examples below:

Direct access to or participation in the community

- Integrated competitive employment
- Adult learning
  - Education
  - Training
- Housing
  - Place
  - Housemates
  - Usability
- Civic Involvement
  - Voting
  - Advocacy
  - Committees/leadership
  - Volunteer work
- Recreation
  - Sports
  - Arts
  - Music
  - Community events (e.g., art in the park, parades, block parties)
- Navigating the community/accessing community (e.g., transportation)

##### Secondary outcomes

The following dimensions of community participation are considered secondary outcomes.

Dimensions of community participation

- Increased self‐determination
  - Autonomy
  - Self‐advocacy
- Improved health
  - Physical
  - Mental
- Improved quality of life
- Increased family support/activities in the home
  - Caregiving
  - Supporting children/parenting
  - Household chores/care
- Social networking
  - Friendships
  - Relationships (e.g., boy/girlfriend, spouse)
  - Church/religious activities

#### 5.1.6 Duration of follow‐up

Studies were considered for inclusion in the review if they measured outcomes beyond an immediate posttest. All studies meeting the inclusionary criteria and reporting follow‐up data were analyzed for treatment effects for the variables reporting appropriate data for analysis. The same criteria and statistical procedures were used for follow‐up conditions as for the primary intervention data. In this manuscript, follow‐up data are reported separately from the primary intervention data.

#### 5.1.7 Types of settings

The criteria for types of settings for the interventions were as follows.

- Interventions that were applied in a community‐based setting were included.
- Research settings at Universities or at community‐based settings that were devised specifically for the study to accommodate persons with a disability were included.
- Interventions conducted in in‐patient institutions, hospitals, or other restrictive residential settings were excluded.

### 5.2 Search methods for identification of studies

#### 5.2.1 Electronic searches

##### Peer‐reviewed literature

Our team includes a University of Kansas Associate Faculty Librarian with experience conducting systematic and scoping reviews (Peterson‐Besse et al., 2014; White et al., 2013), and who collaborated with study researchers to determine appropriate search strategies and conducted the electronic searches. In addition, an advisory team, a subgroup of the Scientific‐Consumer Advisory Panel (SCAP) for the RRTC/PICL project consisting of three outside scientists and one consumer advocate, advised when the study researchers had unresolved questions about definitions, search criteria, and other study related issues.

Database selection is important because of the need to have the most comprehensive, but least duplicative, results as possible. Therefore, our first step was to identify relevant articles and journals in which the researchers and advisory team predicted the most relevant studies would appear. We identified the following fields of research where we might find literature and journals associated with each topic.

- Employment & Rehabilitation
  - Disability and Rehabilitation
  - Journal of Vocational Rehabilitation
  - Journal of Applied Rehabilitation Counseling
  - Rehabilitation Research, Policy, and Education
  - Journal of Occupational Rehabilitation
  - Journal of Labor Policy
  - Journal of Rehabilitation
  - Rehabilitation Counseling Bulletin
- Community psychology
  - Journal of Counseling Psychology
- Medicine/public health
  American Journal of Public Health
- Aging
  - The Gerontologist
- Disability
  - Disability and Society
  - Research in Developmental Disabilities
  - Intellectual and Developmental Disabilities
  - Disability and Health Journal
  - Journal of Intellectual and Developmental Disability
  - Journal of Intellectual Disabilities
  - Journal of Applied Research in Intellectual Disabilities
  - Autism
  - Journal of Intellectual Disability Research
  - Journal of Disability Policy Studies
  - Research and Practice for Persons with Severe Disabilities
  - Focus on Autism and Developmental Disabilities
  - Education and Training in Developmental Disabilities

The next step was to identify which electronic databases index the journals by searching Ulrich's Periodical database. We searched the databases with the largest number of identified journals indexed. We also ensured that all identified journals listed above were represented within the databases that we reviewed. After reviewing 15 databases and one publisher journal package, we selected three databases for initial electronic searching: PubMed, Web of Science, and PsycInfo.

The databases were selected based on guidelines in the Cochrane Handbook about database selection, including the necessity of searching of PubMed, and the inclusion of both a general bibliographic and a subject‐specific database (Higgins & Green, 2011). The search strategies were informed by previous systematic reviews (Peterson‐Besse et al., 2014; White et al., 2013). The goal was to ensure that all types of disabilities were included in the search results, while excluding irrelevant results (see Section 5.1.2) In addition, each database has a specific search strategy because of the different controlled vocabulary and search mechanisms. Database‐provided subject and/or classification limiters were utilized to minimize an excessive number of results since our review search was broad. In particular, the intervention portion of the search was extensive. We developed a spreadsheet with terms related to persons with disabilities that are likely to appear in applicable studies (e.g., sensory disorders, hearing loss, mobility limitation, vision disorders). The search was conducted using terms related to disability and intervention. Keywords included the following:

- Community
  - Participation
  - Living
  - Access
  - Community‐based

- Intervention
- Training
- Multifaceted
- Supports and services
- Intervention outcomes (see list in definitions for outcomes keywords)

Detailed search strategies for all three databases can be found in Supporting Information Appendix A.

The results from all three searches were combined, exported, and deduplicated using the reference management software EndNote.

##### Gray literature

Two additional databases, ProQuest Dissertations and Theses Global and PolicyFile, were searched to identify potential relevant gray literature. Similar intervention and disability searches were implemented, and then limited by year and language in both databases. The results were exported into EndNote for review.

#### 5.2.2 Publication bias

Publication bias occurs in systematic reviews when the included studies do not properly represent the population of studies in a given field. This can happen when studies are not published, thus not being eligible for the population of studies, due to small or null effects (Rothstein, Sutton, & Borenstein, 2005). Because published studies may be more likely to have large effects than unpublished studies, only including published studies may lead to an overestimation of the treatment effect when synthesizing effect sizes in a meta‐analysis.

To investigate the potential impact of publication bias in the present review, we used appropriate measures such as the funnel plot in combination with Egger's regression test for funnel plot asymmetry (Egger, Smith, Schneider, & Minder, 1997; Light & Pillemer, 2004).

### 5.3 Data collection and analysis

#### 5.3.1 Selection and screening of studies

After the results from all three searches were combined, the abstracts of the articles were exported to EndNote. A team of three coders screened studies for inclusion/exclusion at three stages, Stage 1: abstract, Stage 2: full‐text for initial criteria, and Stage 3: full‐text for methodology. We selected 15 studies that met criteria for inclusion in the study and were of “acceptable” (13) or “high” (2) methodological quality for inclusion in the meta‐analysis.

##### Stage 1: Abstract

First, we each read, reviewed, and discussed 10 practice articles to test our inclusionary criteria. We practiced applying the inclusionary criteria on this first set of articles to work to come to consensus about the application of the inclusionary criteria and the definitions of key terms within those criteria. This helped to clarify our understanding of the criteria and how they were applied. This also prompted several clarifying questions posed to the advisory committee regarding terms related to setting, outcomes, and disability.

Once we had clarity on those terms and discussed them as a team, we then tested these revised inclusionary criteria on 10 additional practice articles. Our team of three researchers, reviewed these articles, primarily by abstract and occasionally looking deeper into the methods section, to determine appropriateness for the sample. First, we reviewed the articles independently and applied the revised inclusionary criteria. We then came together and discussed each article in the sample and came to consensus on the application of the criteria, clarifying terms as needed.

Next, we applied the initial screening criteria independently to a new set of five practice articles and achieved 60% reliability/agreement among the three researchers with that sample. This initial 60% reliability/agreement reflected the need for the research team to do additional work in clarifying definitions and providing examples and nonexamples as we encountered interventions (many that were medical in nature) and populations (e.g., hoarders, people with diabetes or who had experienced a stroke but had no specified disability) within the literature that we had not originally considered. It was during this time that definitions were narrowed with consultation with the advisory committee and more exclusions were identified through discussion and consensus building. What we thought were clear definitions initially, we found complicated by the literature that we encountered.

After discussion regarding the discrepancies in definitions and decisions on the first set of articles, we applied the revised screening criteria independently to another set of five practice articles and achieved 80% reliability/agreement among the three researchers in the application of the criteria to that sample. During the review of these practice articles, it became clear that since the reviewers had different backgrounds with regard to disability, we needed to work collaboratively in the review process to ensure consistency in decisions. So, initial measures of independent review were not meaningful ultimately, except that they illustrated the need for collaborative decision‐making.

We split up the combined results (4,738 articles) from the systematic searches of the databases previously described among the three researchers. The review team researchers each screened a set of abstracts to determine whether initial inclusionary and exclusionary criteria were met, and decisions for each article were recorded. A total of 551 of the 4,738 articles (12%) were initially reviewed by two researchers. This paired review was conducted when it was determined that there was a need for input from another researcher with content knowledge expertise. Decisions for those articles were made jointly by the two reviewers. Once the researchers had coded the articles, based on the abstract review, the research team reviewed the decisions for each article together to ensure consistency across application of criteria and screening decisions.

Throughout the screening process, the lead researcher was consulted with questions regarding criteria, corresponded with the advisory committee, and reviewed the decisions. The initial inclusionary criteria were as follows:

- Original research involving testing an intervention
- Interventions must be applied in community‐based settings
- Intervention must target one or more community participation outcomes
- Intervention targets persons with a disability/ies for at least a portion of the
- Target sample—with identifiable outcomes for the disability sample

In this process, we removed 4,552 articles from the search results, leaving 186 articles on which to examine the body of the text to determine inclusivity in the review.

##### Stage 2: Full‐text for initial criteria

We reviewed the full‐text of 186 articles to determine if they met the initial criteria for inclusion. The decision for advancing the retrieved abstracts to the full‐text stage was made based on whether the abstract met the initial criteria and whether additional information was needed to make a decision. Due to the complex definition of “multifaceted intervention” and the diverse backgrounds of the researchers on the team, coding for each study in the remaining 186 articles slated for a full‐text review was completed jointly by at least two researchers completing a coding sheet in Excel that tracked the four bullet points above for each full‐text article that was reviewed, including the team agreed‐upon final decisions for determination of inclusion in the next phase of review. The application of the criteria and decisions were discussed, and consensus was reached whenever there were disagreements regarding the inclusion of the article. Since all decisions were made in collaboration based on the review and discussion by at least two researchers, interrater reliability was not computed as there were no independent determinations made. When a consensus was reached to include the article in the review, the article was designated as one that would be among those reviewed for methodology. After the full‐text review, we had 37 articles that met initial criteria for inclusion.

##### Stage 3: Full‐text for methodology

The third and final stage of review conducted was on the quality of the studies. We used an adapted version of the NTACT Quality Indicator Checklists for Group Experimental studies (see, https://transitionta.org/system/files/effectivepractices/Quality%20Indicator%20Checklist_Group_11‐04‐16%20(2).pdf?file=1&type=node&id=1131&force=). We did not use the initial criteria included in the checklist as we had already applied our own initial inclusionary criteria, and we adapted the language, which specifically targeted studies in the field of transition services, to be generic to our purpose. We retained the all quality review criteria (1–19) and the requirements for overall quality determination. We inserted the criteria into an excel sheet and to track the decisions for all 19 criteria as well as final decisions for determination of inclusion in the review. Two reviewers read the full text of all 37 articles, applied the quality checklist, held discussions to regarding the ratings, and made a joint determination on inclusion based on the ratings. When the reviewers disagreed regarding the application of the criteria, they reached a consensus on each rating through discussion. All quality ratings were assigned based on the joint review and discussion of at least two researchers. After conducting the full‐text review for quality of methodology, 15 articles remained to be included in this meta‐analysis.

#### 5.3.2 Data extraction and management

At least two reviewers independently extracted data for all eligible studies and then reviewed and compared the data for accurate entry. They used a coding sheet to gather data from each article. When two or more study samples provided sufficient information to permit effect size calculations, we conducted random‐effects meta‐analyses to synthesize effects across studies.

#### 5.3.3 Assessment of risk of bias in included studies

In the final full‐text review stage, we assessed each included study for methodological quality using as the risk of bias for such characteristics as reporting, internal validity bias, selection bias, external validity bias, and attrition bias using an adapted version of the NTACT Quality Indicator Checklists for Group Experimental studies.

#### 5.3.4 Measures of treatment effect

We made all calculations using the metafor and robumeta packages in R to synthesize, compute, compare, and determine variation in effect sizes and treatment effects across the reviewed studies (Fisher, Tipton, & Zhipeng, 2017; Viechtbauer, 2010). The calculation of effect sizes account for the variety of measures used to assess intervention effects by calculating and analyzing all data using the standardized mean difference converted to Hedges' *g*. For those studies reporting *F* test, *t* test, or *p* values rather than means and standard deviations, effect sizes are calculated using appropriate conversion conventions in R.

In order to provide a substantive interpretation of the intervention effects and retain effect size independence, the effect sizes from the individual studies are aggregated within and across studies using only same or conceptually similar outcomes. For example, for studies reporting hours worked, salary, job tenure, these data are classified as employment outcomes with the combined treatment effect on employment aggregated across other studies reporting employment outcomes. Categories of measured community participation outcomes include employment, quality of life, mental health, adult learning, activities of daily living, autonomy, independent living, social skills, community activities, and housing. Different outcomes categories unrelated to the community participation outcomes of interest were not analyzed.

#### 5.3.5 Unit of analysis issues

The unit of analysis was determined based on the presentation of outcome data. For those studies presenting participant (e.g,. student, patient, client, consumer) data the unit of analysis was at the individual level. For those studies presenting at a group level (e.g., center, state) the level of analysis was conducted at the group level.

#### 5.3.6 Dealing with missing data

Attrition was calculated for each study and an evaluation was conducted to assess overall quality of the study. No included study was eliminated from the analysis due to missing data.

#### 5.3.7 Assessment of heterogeneity

Heterogeneity analysis was conducted for participant, intervention, and outcome characteristics. In light of the fact that multiple effect sizes may be attributable to sampling error, a random effects model and the associated inverse variance weight at the 95% confidence level was used for all analysis. The random effects model provides for an assumption of population variation from which the sample is drawn and calculates the impact of the effect size by estimating the parameters of that population.

#### 5.3.8 Assessment of reporting bias

The assessment of reporting bias was accomplished via an assigned NTACT rating as *high quality*, *acceptable quality*, or *did not meet quality*. The rating of *high quality* required that all items be positively accounted for in the text, while *acceptable quality* required that items 1–6, 9, 10, 12, 16, and 17 be present. A rating of *did not meet quality* required that one or more of the *acceptable quality* items be absent.

#### 5.3.9 Data synthesis

Data extracted from all included studies reported continuous outcomes, the effect size data was synthesized using outcome means and standard deviations when available. For those studies reporting *F* test, *t* test, or *p* values rather than means and standard deviations, effect sizes were calculated using appropriate conversion conventions provided by the metafor package in R (Viechtbauer, 2010). No studies reported dichotomous data. When two or more studies using a common group comparison (e.g., T vs. C or T_1_ vs. T_2_) were reported a meta‐analysis was conducted as provided by the metafor package in R (Viechtbauer, 2010). The meta‐analysis used a correlated effects model with small‐sample size correction for analysis of effect sizes across studies aggregated on the basis of a common outcome. To account for potential issues of dependency within studies, Robust Variance Estimation was used to estimate the standard errors.

#### 5.3.10 Subgroup analysis and investigation of heterogeneity

Subgroup analysis was planned for (a) types of direct access to community participation, (b) dimension of community participation, (c) length of intervention, (d) place of intervention, (e) type of outcome measure, and (f) disability type. However, because of an insufficient number of studies, analysis was only conducted for employment and length of intervention.

#### 5.3.11 Sensitivity analysis

A one‐removed procedure was conducted as a sensitivity analysis to assess the strength of a single study effect size on the overall effect size for those outcomes in which multiple studies contributed to a common outcome. No other sensitivity analysis was appropriate due to the small number of studies or represented in the measured outcomes.

## 6 RESULTS

### 6.1 Description of studies

#### 6.1.1 Results of the search

The electronic databases searches yielded 4,738 potentially relevant documents for review. During the review, we collected an additional five articles identified through ancestral searching/specific references to relevant articles during the review of full texts for a total of 4,743.

These documents were filtered for duplicates while conducting the searches, and we used Endnote (a bibliographic management software) to sort and identify further duplicates prior to beginning the review of the search results. Of these 4,743 documents we reviewed, an additional 18 were duplicate hits, which we eliminated from further consideration. We reviewed the titles and abstracts of the 4,743 documents to determine potential relevance, excluding 4,556 due to irrelevance to the review, leaving 186 articles on which to examine the body of the text to determine inclusion in the review. At the initial review stage, two researchers reviewed 12% of the manuscripts by title and abstract to check for alignment among researchers in application of the inclusionary criteria and to resolve any uncertain determinations.

**Figure 3:**
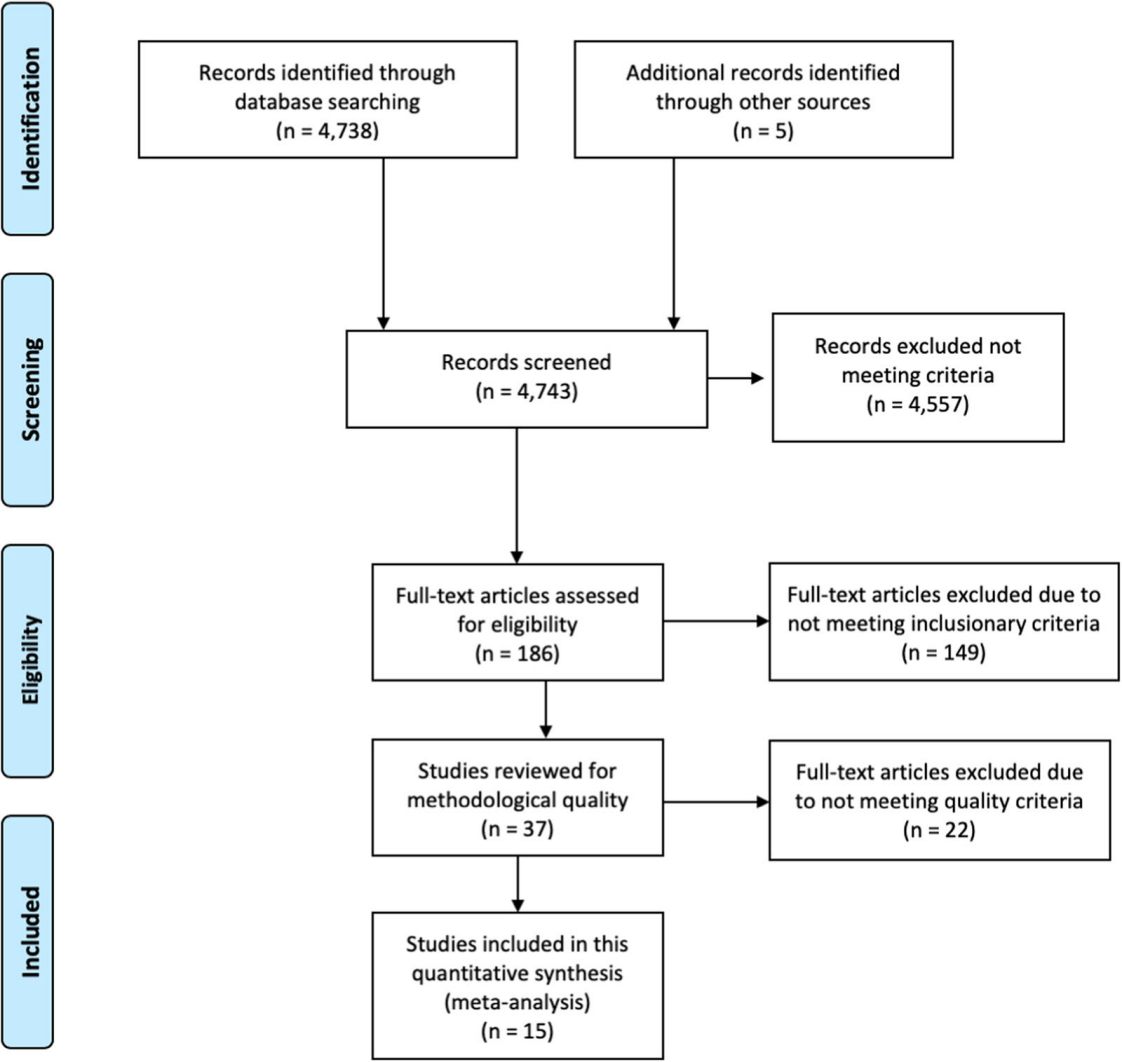
Prisma flow‐chart

Next, we obtained and reviewed 186 full‐text documents, meeting weekly to discuss the articles and come to consensus on the determination of inclusion in the meta‐analysis. Of the 186, 37 full‐text documents were determined to have content appropriate for the review. We then reviewed the remaining 37 manuscripts for methodological quality. We excluded 22 studies due to ineligible or poor‐quality study design. Fifteen studies (peer‐reviewed manuscripts) met all eligibility criteria and were included in the meta‐analysis. Figure 3 illustrates the flow of studies through the systematic review process.

#### 6.1.2 Included studies

An examination of the full texts of 186 potentially relevant studies resulted in 15 studies being included in the meta‐analysis (see Table 1), representing 3,296 treatment participants and 3,782 control or comparison participants. All included studies employed a multifaceted intervention with participants with disabilities. We conducted a meta‐analysis of the impact of multifaceted interventions on the following community participation outcomes: employment, quality of life, mental health, adult learning, activities of daily living, autonomy, independent living, social skills, community activities, and housing.

**Table 1:** Scoring for all included studies using the NTACT to assess risk of bias

		Participant				Intervention and comparison				Outcome measures							Data analysis		
	Item	1	2	3	4	5	6	7	8	9	10	11	12	13	14	15	16	17	18
Author and year	Rating	A	A	A		A	A			A	A		A				A	A	
		H	H	H	H	H	H	H	H	H	H	H	H	H	H	H	H	H	H
Tsemberis and Eisenberg (2000)	Acceptable quality	+	+	+	+	+	+	+	+	+	+	+	+	+	+	−	+	+	+
Birk et al. (2004)	Acceptable quality	+	+	+	+	+	+	−	+	+	+	+	NA	+	−	−	+	+	+
Cook et al. (2005)	Acceptable quality	+	+	+	+	+	+	+	+	+	+	+	NA	+	+	−	+	+	+
McGurk et al. (2007)	Acceptable quality	+	+	+	+	+	+	−	+	+	+	+	NA	−	+	−	+	+	+
Onor et al. (2007)	High quality	+	+	+	+	+	+	+	+	+	+	+	NA	+	+	+	+	+	+
Bell et al. (2008)	Acceptable quality	+	+	+	+	+	+	+	+	+	+	+	+	−	+	+	+	+	+
Fleming et al. (2009)	Acceptable quality	+	+	+	+	+	+	−	+	+	+	+	+	−	+	+	+	+	+
Gutman et al. (2009)	Acceptable quality	+	+	+	+	+	+	−	+	+	+	+	+	+	+	−	+	+	+
Kurz et al. (2009)	Acceptable quality	+	+	+	+	+	+	−	+	+	+	+	NA	−	+	−	+	+	+
Mirza and Hammel (2009)	High quality	+	+	+	+	+	+	+	+	+	+	+	NA	+	+	+	+	+	+
Tsang et al. (2009)	Acceptable quality	+	+	+	+	+	+	+	+	+	+	+	+	+	−	+	+	+	+
Gimm et al. (2011)	Acceptable quality	+	+	+	+	+	+	+	+	+	+	+	+	+	+	−	+	+	+
Szanton et al. (2011)	Acceptable quality	+	+	+	+	+	+	−	+	+	+	+	+	+	+	+	+	+	+
Ferguson et al. (2012)	Acceptable quality	+	+	+	+	+	+	−	+	+	+	‐	NA	‐	+	+	+	+	+
Twamley et al. (2014)	Acceptable quality	+	+	+	−	+	+	+	+	+	+	+	+	+	−	−	+	+	+

*Note*: A—item is required for rating the manuscript as “acceptable quality” for inclusion in the review. H—item is required for rating the manuscript as “high quality” for inclusion in the review.

##### Participants

The studies included in the meta‐analysis had participants who were 18 years or older, identified as having one or more disabilities, and who had exited the secondary education/high school setting and services.

The participants in the included studies were representative of only four disabilities (mental health, TBI, developmental disability, and aging‐related disabilities). Seven studies (Bell, Zito, Greig, & Wexler, 2008; Cook et al., 2005; Ferguson, Xie, & Glynn, 2012; Gutman, Kerner, Zombek, Dulek, & Ramsey, 2009; McGurk, Mueser, Feldman, Wolfe, & Pascaris, 2007; Tsang, Chan, Wong, & Liberman, 2009; Tsemberis & Eisenberg, 2000) targeted people experiencing mental health challenges. Two studies (Fleming, Kuipers, Foster, Smith, & Doig, 2009; Twamley, Jak, Delis, Bondi, & Lohr, 2014) targeted people with TBI. Four studies (Birk et al., 2004; Kurz, Pohl, Ramsenthaler, & Sorg, 2009; Onor et al., 2007; Szanton et al., 2011) targeted aging populations experiencing disability as measured through activities of daily living and instrumental activities of daily living. One study (Mirza & Hammel, 2009) targeted people with developmental disabilities, and one study (Gimm, Ireys, Gilman, & Croake, 2011) did not report the specific disability of the target population.

##### Location of the studies

There were five countries (i.e., United States, China, Germany, Italy, and Australia) represented in the included studies, with the majority of studies (10) coming from the U.S. Two studies came from Germany and one each from China, Italy, and Australia.

##### Study design

Of the 15 included studies, five were quasiexperimental studies (i.e., Birk et al., 2004; Ferguson et al., 2012; Fleming et al., 2009; Kurz et al., 2009; Tsemberis & Eisenberg, 2000) and 10 were RCTs (i.e., Bell et al., 2008; Cook et al., 2005; Gimm et al., 2011; Gutman et al., 2009; McGurk et al., 2007; Mirza & Hammel, 2009; Onor et al., 2007; Szanton et al., 2011; Tsang et al., 2009; Twamley et al., 2014).

##### Intervention description

All studies employed multifaceted interventions designed to impact outcomes related to community participation. We defined *multifaceted interventions* as interventions that address two or more individual (changing something about the person—enhancing skills/knowledge, changing behavior, changing perceptions/attitudes) or environmental characteristics (changing something about the people, places, or things in the environments in which the person interacts) in different domains (e.g., social skills/inclusion, financial resources/management, physical health, mental health, employment, transportation, adult learning, health care). Many of the multifaceted interventions used with these populations included a cognitive coaching component that supported improved executive functioning.

##### Outcomes description

We defined *community participation outcomes* as outcomes that are chosen by/desired by the person with a disability, occur in the community (i.e., integrated with people without disabilities), and are reflective of (a) direct access to or participation in the community or (b) dimensions of community participation (i.e., outcomes for which there is a research base linking them to community participation). We identified the following as primary community participation outcomes that offered direct access to or participation in the community: integrated competitive employment, adult education, housing, civic involvement, recreation, navigating the community (e.g., transportation or mobility training). We identified the following as secondary community participation outcomes that we described as dimensions of community participation for which there is a research base linking them to direct access community participation outcomes: self‐determination/autonomy, physical or mental health, quality of life, activities in the home (e.g., caregiving, household chores), social networking.

Included studies examined the outcomes of employment (Bell et al., 2008; Cook et al., 2005; Ferguson et al., 2012; Fleming et al., 2009; Gimm et al., 2011; McGurk et al., 2007; Mirza & Hammel, 2009; Tsang et al., 2009; Twamley et al., 2014), quality of life (Szanton et al., 2011; Twamley et al., 2014), mental health (Birk et al., 2004; Kurz et al., 2009; Onor et al., 2007), adult learning (Gutman et al., 2009), activities of daily living (Birk et al., 2004; Kurz et al., 2009; Onor et al., 2007; Szanton et al., 2011), autonomy (Birk et al., 2004), independent living (Fleming et al., 2009), social skills (Fleming et al., 2009; Gutman et al., 2009), community activities (Fleming et al., 2009), and housing (Tsemberis and Eisenberg, 2000). With the variety of outcomes across studies, quantitative synthesis was challenging because the measures varied across studies even within the same outcome. Supporting Information Appendix B provides a complete list of the individual outcomes and their associated effect size is provided for each included study.

#### 6.1.3 Excluded studies

Studies that were excluded from the analysis may have employed interventions that had multiple components (i.e., multifaceted), but the interventions did not focus on two different domains. There were 149 excluded studies due to ineligible interventions or ineligible study design. Among studies with ineligible designs, single population studies without comparison groups were common. We also excluded studies because the participants did not meet criteria either due to age (e.g., younger than 18) or the described disability was excluded from our defined population (e.g., obesity, diabetes, hoarding, phobias, substance abuse, suicidal tendencies, HIV/AIDS, arthritis, stroke with no other reported disability).

### 6.2 Risk of bias in included studies

Risk of bias for the included studies was assessed in applying the adapted NTACT quality assessment tool to the 15 included studies. As shown in Table 1 below, two studies met the *high quality* rating requirements and the remaining 13 studies met the *acceptable quality* requirements. Specific items included in the quality rating were judged as present (+), not present (−), or not appropriate (NA). The NA rating was used only on item 12 as per NTACT directions. As indicated earlier in this review, the standard for a high quality rating required a study to achieve a positive rating for all 18 items of NTACT while an acceptable quality rating required for items 1, 2, 3, 5, 6, 9, 12, 16, and 17.

#### 6.2.1 Publication bias

**Figure 4:**
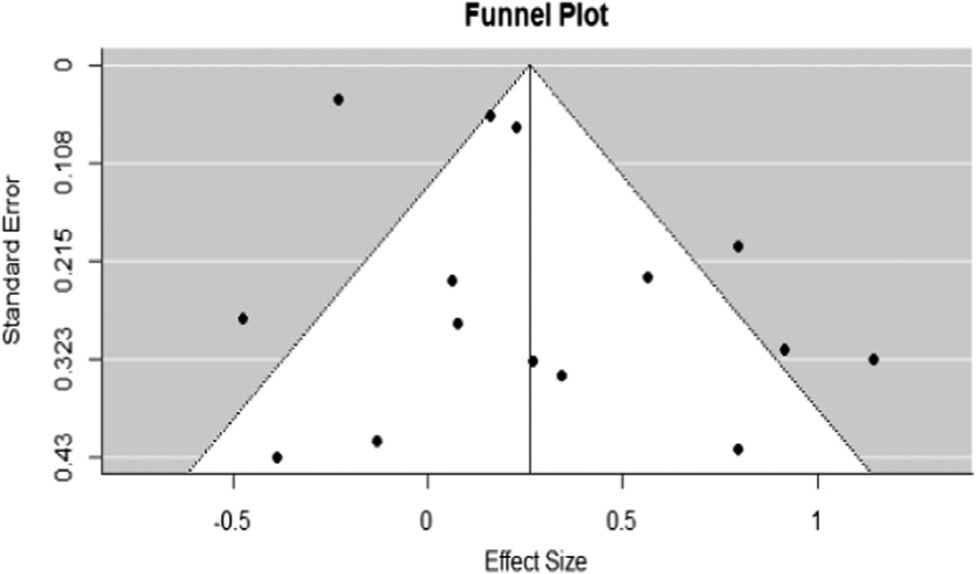
Funnel plot

Examining the funnel plot in Figure 4 we see that studies, identified by a single point in the plot, are generally evenly distributed while roughly forming the funnel shape. This is an initial indicator that it is likely publication bias is not significantly impacting the overall results of this systematic review.

Results from the Egger's regression test further confirm this notion (*t* = 0.724, df = 13, *p* = .482). With a nonsignificant result from the Egger's regression test aligning with the visualization of the funnel plot, we are confident that the results of this systematic review are not significantly impacted by publication bias.

### 6.3 Synthesis of results

Data synthesis for this review was conducted using R, first by calculating individual outcome effects for each study using the metafor package, then aggregating and meta‐analysing the effects using the robumeta package across the included studies (Fisher et al. 2017; Viechtbauer, 2010). All results are reported as Hedges' *g* with corresponding variances. Moderator analyses were accomplished where appropriate through a subgroup analysis procedure and the one‐study removed procedure to assess potential study bias. These analyses are organized according to the primary treatment outcomes of employment, activities of daily living, adult learning, mental health, quality of life, autonomy, social skills, community activities, independent living skills, and housing.

For each outcome category, a subgroup moderator analysis was conducted for those characteristics of interest that provided at least two studies representing each subgroup.

#### 6.3.1 Assessment of heterogeneity

In addition to the overall effect size, we report two measures of heterogeneity to assess the level of consistency across studies included in the meta‐analysis. First, we report *I* ^2^, which measures the percentage of between‐study variance attributable to true heterogeneity and not sampling error (Borenstein, 2009; Higgins & Thompson, 2002). Next, we report *τ* ^2^, which is a measure of the dispersion of true effect sizes between studies (Borenstein, 2009).

The *I* ^2^ (*I* ^2^ = 92.01%) indicates that 92.01% of the between‐study variance is due to true heterogeneity. According to Higgins et al. an *I* ^2^ of 25.00 is considered low, 50.00 moderate, and 75.00 high (Higgins, Thompson, Deeks, & Altman, 2003). Because of the high degree of heterogeneity in the present meta‐analysis, it follows that moderator variables impact the result. Similar to what was drawn from the *I* ^2^ result, the *τ* ^2^ (*τ* ^2^ = 0.182) indicates a high degree of heterogeneity.

Thus, the following analyses and results are presented as a refined assessment of treatment effects as independent measures for design, participant, treatment, and outcome characteristics for each of the observed outcomes. These analyses are organized by outcome according to the design, and treatment classifications described earlier in this paper.

As we discuss the studies analysed below, we use “Experimental” to refer to the populations that are receiving the multifaceted intervention. We use “Control” or “Comparison,” as appropriate, to refer to the populations to which the outcomes of the Experimental group receiving the multifaceted intervention is compared.

#### 6.3.2 Employment outcome

##### Design characteristics

A total of five studies measured employment outcomes for treatment effects. All of these studies reported an RCT Experimental (RCT Exp) versus Control (Ctl) group design and represent combined employment outcomes (e.g., number of jobs, length of employment, wages, etc.). Table 2 is a summary of the meta‐analysis for the effect sizes associated with the employment outcomes for each study contributing to the overall effect. These data suggest that the multifaceted interventions did not have a significantly positive effect on the measured employment outcomes (*g* ^+^ = 0.444, 95% confidence interval [CI] [−0.061, 0.949], *p* = .085) when compared to a nontreated control group.

**Table 2:** Employment outcome effect sizes for RCT Exp versus Ctl design

Studies	Hedges' *g*	Lower limit	Upper limit	*p* value
McGurk et al. (2007)	0.917	0.303	1.531	.003
Bell et al. (2008)	0.109	−0.349	0.568	.639
Mirza and Hammel (2009)	0.565	0.009	1.031	.018
Tsang et al. (2009)	0.891	0.497	1.248	.000
Gimm et al. (2011)	−0.121	−0.200	−0.042	.003
Overall effect	0.444	−0.061	0.949	.085

Abbreviations: Ctl, control; RCT Exp, randomized controlled trial experimental.

Another design factor of interest was the method of data analysis. Specifically, we were interested in comparing the studies using and intent‐to treat (ITT) analysis and the treatment‐on‐the‐treated (TOT) methods of analysis. These data reveal a statistically significant difference between the two methods of analysis (Q between *p* = .000) with the larger effects associated with the TOT method of analysis. These data suggest the possibility of larger effects size associated with the TOT analysis. A summary of this analysis is presented in Table 3.

**Table 3:** Employment outcome associated with method of analysis in the Exp versus Ctl design

Method of analysis	Studies	Hedges' *g*	Lower limit	Upper limit	*p* value
ITT	Bell et al. (2008)	0.109	−0.349	0.568	.639
ITT	Gimm et al. (2011)	−0.121	−0.200	−0.042	.003
	Combined	−0.115	−0.192	−0.370	.004
TOT	McGurk et al. (2007)	0.917	0.303	1.531	.003
TOT	Mirza and Hammel (2009)	0.565	0.009	1.031	.018
TOT	Tsang et al. (2009)	0.891	0.497	1.248	.001
	Combined	0.786	0.516	1.056	.001

Abbreviations: Ctl, control; Exp, experimental.

No other design related factor involved at least two studies thus no additional moderator analyses were conducted for design characteristics.

Follow‐up assessment of employment outcome treatment effects was conducted for two studies (Bell et al., 2008; Tsang et al., 2009). Bell reported follow‐up measurements at 12 months postintervention while Tsang reported follow‐up measurements at 11‐ and 15‐months postintervention for the experimental versus control group comparisons. Due to the differences in measurement times, no aggregation of results was deemed appropriate. Individual study results of the treatment effects revealed a nonsignificant treatment effect for the Bell study (*g* = 0.410, 95% CI [−0.420, 0.502] *p* = .862) and significant treatment effect for the Tsang study at 11 months (*g* = 1.104, 95% CI [1.224, 2.099] *p* = .000) and at 15 months (*g* = 1.001, 95% CI [0.579, 1.423], *p* = .0.000).

##### Treatment characteristics

The treatment characteristics identified across the 15 studies included the type of treatment, length of treatment program, length of treatment sessions, and number of treatment sessions. The only treatment characteristic, in those studies looking at employment outcomes, that provided appropriate effect sizes for a subgroup analysis was the length of treatment feature. As seen in Table 4, the 1–10 weeks studies (McGurk et al., 2007; Mirza & Hammel, 2009) produced a *g*+ = 0.694 while the 20+ weeks studies (Bell et al., 2008; Gimm et al., 2011; Tsang et al., 2009) yielded a *g*+ = 0.277. The resulting combined effect size (*g* = 0.587) was found to be statistically nonsignificant (Q 1, *p* = .267) suggesting that the difference between the two lengths of interventions were not significantly different but that the overall combined treatment effect was statistically significant (*p* = .000).

**Table 4:** Employment outcome associated with the length of the treatment program

Length of treatment	Study	Hedges' *g*	Lower limit	Upper limit	*p* value
1–10 weeks	Mirza and Hammel (2009)	0.565	0.009	1.031	.018
1–10 weeks	McGurk et al. (2007)	0.917	0.303	1.531	.003
1–10 weeks effect		0.694	0.322	1.065	.000
20+ weeks	Bell et al. (2008)	0.109	−0.349	0.568	.639
20+ weeks	Tsang et al. (2009)	0.891	0.497	1.248	.000
20+ weeks	Gimm et al. (2011)	−0.121	−0.200	−0.042	.003
20+ weeks effect		0.277	−0.355	0.909	.390
Overall effect		0.587	0.267	0.907	.000

No other treatment related factors in the area of employment provided the required two or more studies representing the characteristic of interest for further analysis. Thus, no additional moderator analyses were conducted.

One study (Tsang et al., 2009) compared two multifaceted interventions against each other as well as a Control group. One experimental group (Exp1) was an Integrated Support Employment (ISE) approach, while the second experimental group (Exp2) received an Individual Program Support (IPS) approach to intervention. The control group received neither intervention with the results reported in Table 4 above. All of the outcomes were employment related for both Exp1 (ISE) and Exp2 (IPS) groups with the first postintervention measurement taken at seven months and follow‐up data taken at 11 and 15 months. At each time point a statistically significant treatment effect size emerged in favor of the ISE approach: at 7 months (*g* = 0.468, 95% CI [0.087, 0.849], *p* = .016); at 11 months (*g* = 0.568, 95% CI [0.153, 0.961], *p* = .006; at 15 months *g* = 0.656, 95% CI [0.406, 0.907], *p* = .001).

A total of three studies (Cook et al., 2005; Ferguson et al., 2012; Fleming et al., 2009) utilized an Experimental versus Comparison (Exp vs. Comp) design to assess treatment effects for employment outcomes. Ferguson and Fleming reported an RCT based Exp versus Comp design while Cook used QED based Exp versus Comp design. A summary of the effect size associated with each study and their aggregated effect size is presented in Table 5.

**Table 5:** Employment outcome effect sizes for Exp versus Comp

Study	Design	Hedges' *g*	Lower limit	Upper limit	*p* value
Cook et al. (2005)	QED	0.162	0.052	0.272	.004
Fleming et al. (2009)	RCT	−0.699	−1.358	−0.04	.038
Ferguson et al. (2012)	RCT	0.798	−0.046	1.642	.064
Overall effect		0.060	−0.585	0.706	.854

Abbreviations: Ctl, control; Exp, experimental; QED, quasiexperimental design; RCT, randomized controlled trial.

Since Cook used a QED for study construction, it was of interest to determine if there was an overall effect on the Exp versus Comp study design. Thus, a sensitivity analysis was conducted via the one‐study removed procedure revealing a statistically nonsignificant impact on the overall effect size (*g* = 0.025, 95% CI [−1.441, 1.492], *p* = .973) with the Cook study excluded from the aggregation of the overall employment outcomes. Thus, the results in Table 5 indicate that overall, when data for the three studies is aggregated, the multifaceted intervention approach did not result in a significant advantage over the single‐faceted comparison group.

Follow‐up data were available for Ferguson et al. (2012) at 12 weeks and yielded nonsignificant effect size for employment between the two groups (*g* = 0.812, 95% CI [−0.024, 1.648], *p* = .057).

#### 6.3.3 Activities of daily living (ADL) outcome

A total of three studies used an RCT Exp versus Ctl design (Birk et al., 2004; Onor et al., 2007; Szanton et al., 2011) to assess the treatment effects for ADL outcomes. Results of this meta‐analysis revealed an overall nonsignificant treatment effect (*g* = 0.422, 95% CI, [−1.201, 0.358], *p* = .289) (Table 6).

**Table 6:** Activities of daily living outcome effect size, 95% confidence interval, and *p* value

Studies	Hedges' *g*	Lower limit	Upper limit	*p* value
Birk et al. (2004)	−1.078	−1.972	−0.184	.018
Onor et al. (2007)	0.444	−0.495	1.383	.354
Szanton et al. (2011)	0.798	−0.046	1.642	.084
Overall effect	0.422	−1.201	0.358	.289

Similarly, a single study (Kurz et al., 2009) used a QED Exp versus Comp design to assess treatment effects associated with the ADL outcomes for two groups (mild cognitive impairment and dementia). The resulting treatment effect failed to reach statistical significance (*g* = −0.184, 95% CI [−0.948, 0.579], *p* = .636) for either of the two disability groups separately or combined.

#### 6.3.4 Mental health

Mental health outcomes were assessed by three studies (Birk et al., 2004; Kurz et al., 2009; Onor et al., 2007) evaluating the treatment effects for mental health outcomes with an aging population of adults. Birk and Onor compared a multifaceted approach with a no‐treatment control group, while Kurz utilized a multifaceted approach compared to a single‐faceted program. Results of this analysis yielded a combined *g* value of −0.181, (95% CI [1.325,−0.964], *p* = .757. To assess the potential impact of the design, a one‐study analysis indicated that when Kurz was removed from the aggregation, the resulting effects still did not reach significance for the multifaceted group (*g* = −0.738, 95% CI [−1.389, −0.087], *p* = .026) and indicates that the comparison group performed better than the multifaceted group on the measures of mental health.

#### 6.3.5 Adult learning

A single study (Gutman et al., 2009) assessed the treatment effects on outcomes of adult learning for social, task, and interpersonal skill development for participants with psychiatric disabilities using an RCT Exp versus Comp design. The analysis of the treatment effects for this group yielded a positive statistically significant effect (*g* = 1.144, 95% CI [0.806, 1.483], *p* = .001) in favor of the multifaceted intervention over an alternative single‐faceted approach.

#### 6.3.6 Quality of life

Two studies evaluated quality of life for people who have aging‐related disabilities (Szanton et al., 2011) and who acquired a TBI (Twamley et al., 2014) to assess treatment effects associated with participant assessment of quality of life outcomes. Szanton's data were based on an Exp versus Ctl design and yielded a significant treatment effect for QOL measures (*g* = 0.693, 95% CI [0.240, 0.854], *p* = .003) in favor of the multifaceted treatment. Twamley's data were based on an Exp versus Comp design and yielded a nonsignificant treatment effect (*g* = 0.195, 95% CI [−0.464, 0.854], *p* = .561) for the QOL outcomes. Due to the qualitative difference in design, no aggregation of effect sizes was appropriate.

#### 6.3.7 Autonomy

Birk et al. (2004) assessed treatment effects on an autonomy outcome measure using an RCT Exp versus Ctl design. Results of this analysis yielded a *g* value of 0.850, 95% CI [0.023, 1.723;], *p* = .056) indicating a nonsignificant treatment effect.

#### 6.3.8 Independent living, social skills, community activities, and housing

Fleming et al. (2009) was the sole study to assess independent living, social skills and community activities for a group of participants with TBI. Data were drawn from a QED Exp versus Comp design with a self‐report questionnaire used to assess each of the three outcomes. Results of this analysis revealed a significant treatment effect for the comparison groups in the independent living (*p* = .017) and community activities (*p* = .022) outcomes and a nonsignificant treatment effect (*p* = .522) in favor of the comparison group in the social skills outcome as shown in Table 7.

**Table 7:** Outcomes associated with independent living, social skills, and community activities

Outcome	Hedges' *g*	Lower limit	Upper limit	*p* value
Independent living	−0.812	−1.478	−0.146	.017
Social skills	−0.210	−0.850	0.431	.522
Community activities	−0.778	−1.442	−0.115	.022

Tsemberis and Eisenberg (2000) analysed data collected from two different programs serving a homeless mentally ill population in New York City. Tsemberis used an QED Exp versus Comp design in which one program, Linear Residential Treatment, was a public‐funded program designed to provide permanent housing for clients. Pathways Supported Housing was a nonprofit agency that provided services to individuals who were unable to obtain housing through the Linear Residential program. Using a survival analysis, Tsemberis determined that five years later 88% of the Pathways participants and 47% of the Linear participants retained permanent housing. Results of the comparison between the two groups for retention of permanent housing was found to be statistically significant in favor of the Pathways Supported Housing group (*g* = 0.228, 95% CI [0.092, 0.363], *p* = .001).

## 7 DISCUSSION

### 7.1 Summary of main results

We identified, coded, evaluated, and analysed studies that utilized multifaceted interventions to impact community participation outcomes for disabled adults. A total of 15 studies were identified as candidates for the review based on a full text reading of each study by independent reviewers. These studies served as the corpus of data for this review and are reported according to the outcomes identified across all studies.

#### 7.1.1 Employment

Results from those studies reporting on intervention programs to improve employment outcomes, five studies (Bell et al., 2008; Gimm et al., 2011; McGurk et al., 2007; Mirza & Hammel, 2009; Tsang et al., 2009) reported results based on a true experimental design. The meta‐analysis of these studies yielded a nonsignificant intervention impact (*p* = .085). A single study (Cook et al., 2005) also reported results of an intervention impact comparing a multifaceted intervention with a single‐faceted intervention and reported a significantly positive effect for the experimental group (*p* = .004). No overall conclusions based on these data can be offered due to the small number of studies representing the outcome of employment.

One of the design issues that emerged was whether the absence of an overall treatment effect could be due to the method of analysis. The issue of concern was that the more complete analytical method, ITT, has been described as a more accurate representation of the “real world” of intervention in that participants in many settings dropout, do not complete the post assessment, or are otherwise unaccountable for a complete measure of the intervention effect. Alternatively, the TOT approach identifies the treatment effect for participants who complete the entire intervention program including all assessment measures. This distinction can be viewed partly as statement of practicality verses reality, and as such becomes a matter of the nature of the application of the findings.

Those studies using an ITT approach to the data analysis yielded a nonsignificant effect while the studies using a TOT revealed a statistically significant intervention effect for the multifaceted intervention approach (*p* = .001) and a significant difference between the two methods of analysis (*p* = .001). These data suggest the possibility that the less restrictive TOT (*n* = 3) method of analysis may introduce an inherent positive bias resulting in an inflated true intervention effect while the ITT (*n* = 2) approach may more accurately reflect the overall effectiveness of an intervention. In either case, as applied to these data, no definitive interpretation can be drawn due to the small number of studies represented in each group.

A second variable of interest was the length of treatment as a measure of intervention effectiveness. Across the 15 studies the range of the weeks of intervention was 4–105 weeks and were categorized into three groupings: 1–10, 11–20, and 21+ weeks. When this range was applied to the employment related data presented in Table 2, the only time frames of 1–10 and 21+ weeks were represented. Results of the aggregation of studies representing each of these time frames indicates that the shorter intervention time period results in significantly larger program effect in favor of the multifaceted group while the longer intervention produced a small statistically nonsignificant effect (0.390). These data should be cautiously interpreted due to the small number of studies in each time frame as well as the wide variation in the number of weeks represented in the longer time frame. That is, while the 1–10 week studies showed a significantly positive effect, there are no intervening time frames to compare as a measure of intervention effect deterioration between the two time frames. That is, without an intervening measure the trend of the treatment effect is unknown. It might be that the degradation of the intervention effect begins at 21+ weeks.

An additional three studies providing data regarding employment outcomes were included in the review but analyzed separately. These studies used comparison of a multifaceted intervention with a single‐faceted intervention rather than an untreated control group comparison. Two studies used a RCT design (Ferguson et al., 2012; Fleming et al., 2009) for the comparison while Cook et al. (2005) used a QED design. Results of the analysis of these studies suggested that the multifaceted intervention did not result in a significantly positive advantage when compared to the single‐faceted interventions.

#### 7.1.2 Adult learning

Adult learning (Gutman et al., 2009) outcomes were found to demonstrate a positive effect when using a QED design comparing a multifaceted with a single‐faceted intervention. The findings supported the multifaceted group over the comparison group (*g* = 1.144); however, without a no‐treatment control group, no conclusion can be drawn as to the independent effectiveness of the multifaceted intervention. The further potentially confounding factor of different designs makes any confirming statement of treatment effect inappropriate.

#### 7.1.3 Activities of daily living

Three studies (Birk et al., 2004; Onor et al., 2007; Szanton et al., 2011) evaluated multifaceted interventions’ impact on ADL compared to a nontreated control group. Additionally, Kurz et al. (2009) reported a QED designed study reporting a comparison between multifaceted and single‐faceted programs. With the results of all four studies yielding statistically nonsignificant findings in favor of the multifaceted participants, either the usefulness of the outcomes measures or the multifaceted intervention itself are potentially suspect. As stated earlier, these data too must be interpreted with caution due to the small number of studies represented in the analysis.

#### 7.1.4 Mental health

The analysis of the effect of a multifaceted approach for mental health outcomes was represented by three studies, (Birk et al., 2004; Kurz et al., 2009; Onor et al., 2007). The results also yielded a lack of support for the multifaceted approach. Due to the small number of studies available, no conclusion or explanation can be offered for the treatment effects related to mental health.

#### 7.1.5 Quality of life

Quality of Life outcomes were assessed by two studies using different designs for an aging disabled population (Szanton et al., 2011) and a population with TBI (Twamley et al., 2014). Szanton et al. (2011) found a statistically significant impact for quality of life measures (*p* = .003), while the Twamley et al. (2014) study yielded a nonsignificant outcome (*p* = .561). Due to design differences, the aggregation of these data was not conducted. Due to the small number of studies and the design differences, no substantive conclusions can be drawn.

#### 7.1.6 Autonomy

Outcomes associated with measures of autonomy were reported by Birk et al. (2004). Results of this RTC study yielded a large but nonsignificant effect (*g* = 0.850, *p* = .056). While these results approach statistical significance, with data from only a single study, no conclusion or explanation can be drawn.

#### 7.1.7 Independent living, social skills, community activities, and housing

A single study assessed the effects of independent living, social skills, and community activities (Fleming et al., 2009). None of these outcomes provided support for the multifaceted intervention.

Tsemberis and Eisenberg (2000) reported on an assessment of the effects of a multifaceted intervention compared to single‐faceted intervention on housing retention. While a significant effect was found, no conclusions can be drawn from a single study.

## 8 OVERALL COMPLETENESS AND APPLICABILITY OF EVIDENCE

Due to the variety of outcomes assessed under the domain of “community participation” and the limited number of *high quality* RCT research studies available, the evidence presented here has limited applicability and provides minimal support for the efficacy of multifaceted interventions. For this study, “community participation” was defined to encompass a large number of outcomes related to direct access to and dimensions of community participation. However, there were few articles within each outcome meeting the inclusionary criteria and methodological quality standards that could be synthesized, so it is difficult to draw definitive conclusions from the current evidence base. One outcome addressed by the most studies (five) that could be synthesized was “employment,” which included some support for multifaceted interventions. Additional minimal support was identified for the outcomes of adult learning and quality of life.

Finally, the applicability of the results of this meta‐analysis is greatly limited due to the quality of studies available. While we had several RCT research studies (10), only two were identified as *high quality* when evaluated for methodological quality. Without more rigorous research implementation and thorough reporting, there are limitations to the conclusions that can be drawn from this study sample.

### 8.1 Quality of the evidence

The quality of the body of evidence makes synthesizing effect sizes and drawing conclusions difficult. There were a limited number of *high quality* RCT studies, only two out the 10 RCT studies included in the review. Items that were commonly missing from the reporting on the studies included information related to use of fidelity of implementation measures, blinding of data collectors or scorers, and provision of information regarding the criterion and construct validity of the measures used. Missing any of these features in the review of methodological quality resulted in the study being categorized as *acceptable quality*. It was also quite common for articles to be missing reporting on interrater reliability; however, since that is not always appropriate, depending on the methods used, missing reporting on that criteria may have been considered “not applicable” and thus did not affect the categorization of the methodological quality of the study.

In addition, due to the limited number of *high quality* studies (two), the diversity of types of interventions and methods of data collection, and the wide variety of outcomes that are included in “community participation,” meta‐analysis was not possible for some outcomes, allowing only for reporting of the effect sizes of individual studies. There is little practical significance to the findings from the quantitative synthesis and application of it is limited. Even for outcomes where the individual effect sizes were significant, many were single studies only and, therefore, the quality of the evidence dictates that no practical conclusions can be drawn.

In summary, the strengths of these studies were that (a) the majority of the studies were RCT and had randomly assigned participants, (b) the studies were peer‐reviewed and (c) published in academic research journals. Weaknesses of the studies include a lack of (a) blinding among the data collectors/scorers, (b) fidelity of implementation measures, and (c) criterion and construct validity reporting on the measures used.

### 8.2 Limitations and potential biases in the review process

Limitations of this meta‐analysis include the overall quality of the studies (only two *high quality* RCT studies), the small number of studies identified for the various outcomes of interest related to community participation of adults with disabilities, and the lack of research base focused on the implementation and evaluation of multifaceted interventions in the field.

Potential bias may be introduced with regard to the lack of gray literature included in the meta‐analysis, the differences among research team members in knowledge and education regarding disability and best practices in promoting community participation, and language bias. Although we searched two sources of gray literature, we did not find any eligible studies, which may be indicative of the sources searched, biased inclusionary criteria, or reporting bias. Therefore, all studies included in the meta‐analysis were published in peer‐reviewed journals. Bias may also have been introduced due to the lack of a unified background knowledge on the topic of community participation among the members of the research team. To address this, all full text reviews and decisions were made by at least two researchers on the team coming to consensus on the decision of whether an article should be included. Finally, we recognize that language bias is inherent in the review process because we limited our search to only articles written in English.

We used a rigorous search, screening, and data extraction process, which was conducted by at least two researchers at each stage in order to ensure consistent application of the inclusionary and exclusionary criteria and allow identification of all applicable studies. Our search was conducted for studies published through 2016, so the articles represent the research evidence base available in both peer‐reviewed and gray literature at that time.

### 8.3 Agreements and disagreements with other studies or reviews

Although this systematic review had several studies (15 in total), the application of the results is limited due to the small number of articles that could be synthesized for each outcome of interest related to community participation and the minimal support among them for effective multifaceted interventions. There has been little emphasis placed on the implementation of multifaceted interventions, particularly in the research related to community participation of adults with disabilities. In addition, the lack of research lends to an inconsistent conceptualization of what makes an intervention “multifaceted.” As previously discussed, there were no other studies or reviews available that reflected “multifaceted interventions” as we defined it. We found a systematic review of measures of community participation for people with disabilities (Chang, Coster, & Helfrich, 2013) and one targeting the impact of environmental factors on community participation of persons with intellectual disability (Verdonschot, De Witte, Reichrath, Buntinx, & Curfs, 2009). However, neither of these was specific to multifaceted interventions for community participation of people with disabilities. As such, this review, while offering limited support for multifaceted interventions, fills a gap in the research and provides justification for additional research in this area of study.

## 9 AUTHORS' CONCLUSIONS

### 9.1 Implications for policy

The studies included in this systematic review did not directly address policy, but employment and community living for people with disabilities are important policy areas. As such, continued research is needed to more effectively inform and guide future policies that will support community participation for people with disabilities. This review found limited evidence of the effectiveness of multifaceted interventions to support community participation, so future research efforts may need to refine this method or consider other approaches before implications for policy related to the implementation of multifaceted interventions should be considered.

### 9.2 Implications for research

Limited support for the effectiveness of multifaceted over single‐faceted interventions suggests the need for more substantial research to determine effectiveness broadly as well as specifically in relation to community participation of adults with disabilities. The articles reviewed here considered broad community participation outcomes for adults with a variety of disabilities. Perhaps future research that narrows the focus to more specific outcomes for targeted groups of adults with similar disabilities may yield greater insight into the potential effectiveness of multifaceted interventions.

This review noted that the disability populations that were most frequently the target of multifaceted interventions (i.e., mental illness, TBI, and aging‐related disabilities, including dementia and Alzheimer's) tended to need greater supports with issues of executive functioning. Further, the multifaceted interventions for these populations tended to include cognitive coaching as one of the facets of the multifaceted intervention being applied. Therefore, greater research on multifaceted interventions targeting these key populations may provide greater insight into the effectiveness of multifaceted interventions across specific outcomes of interest (e.g., competitive employment).

Additionally, to enhance the number of studies that may be included as a “multifaceted intervention,” future research could consider an adapted definition that allowed for the intervention points to be within the same domain.

## LEAD REVIEW AUTHOR

The lead author is the person who develops and co‐ordinates the review team, discusses and assigns roles for individual members of the review team, liaises with the editorial base and takes responsibility for the on‐going updates of the review.

## ROLES AND RESPONSIBILITIES

- Content:
  - J. M. S. G. has worked in the field of special education for over 25 years. She has experience and education that is cross disability and lifespan. She has 12 years of experience conducting training, technical assistance, and research in areas related to adults with disabilities and community participation, especially with regard to integrated and competitive employment.
  - A SCAP has been established for our broad research which includes the systematic literature review. The role of the Systematic Review SCAP subcommittee is to offer guidance on selection of keywords, inclusion/exclusion criteria, and quality standards and to advise staff on specific issues that arise in conducting the literature review. At the conclusion of the systematic literature review on multifaceted interventions related to community participation, we will convene a meeting of all SCAP members, including the codirector from the HCBS RRTC on Outcome Measurement, to discuss the implications of our findings.
- Systematic review methods: J. M. S. G., C. N., and D. D.‐G.
  - J. M. S. G. conducted a qualitative systematic literature review for her dissertation related to participant direction of supports and services.
  - C. N.—A consultant who has conducted systematic reviews published in Campbell, Cochrane, and refereed publication.
  - D. D.‐G.—A Researcher with American Institutes for Research (AIR), worked with a team to conduct a systematic literature review for The College Board. In 2016, she participated in an 8‐hr workshop sponsored by AIR on conducting Systematic Literature Reviews. In 2017, Debbie participated in a webinar sponsored by QSR International titled: *Accelerating Your Literature Review with NVivo 11 for Windows*.
  - A. M.‐G.—University of Kansas Associate Faculty Librarian with experience conducting systematic and scoping reviews (Peterson‐Besse et al., 2014; White et al., 2013). She has extensive training in: database searching and retrieval, online database interfaces, quantitative and qualitative research methods, and systematic reviews.
  - D. D.—A Researcher Intern with AIR, and current doctoral student at the University of Texas at Austin in the department of Educational Psychology with a focus on Quantitative Methods. Experience working with a team conducting a review of current practice in quantitative meta‐analyses.

- Statistical analysis: C. N.—experience using CMA; D. D.—experience using R.
- Information retrieval: A. M.‐G. is an Associate Librarian at KU who has previously worked with the RRTC/Community Living on two different systematic reviews.
- MLS, Indiana University; MS, Political Science, Illinois State University; BS, Political Science, Illinois State University

## SOURCES OF SUPPORT

This systematic review will be conducted under a grant from the National Institute on Disability, Independent Living, and Rehabilitation Research (NIDILRR grant number 90RT5043‐01‐00). NIDILRR is a Center within the ACL, Department of Health and Human Services (HHS). The contents of this systematic review do not necessarily represent the policy of NIDILRR, ACL, HHS, and you should not assume endorsement by the Federal Government. In addition, AIR provided staffing support (research assistant and consultant) and technical assistance in the completion of the review to meet Campbell standards.

## DECLARATIONS OF INTEREST

There are currently no known conflicts of interest.

## PLANS FOR UPDATING THE REVIEW

For the RTC/PICL, we plan to use the completed review to inform design and development of multifaceted intervention research in the remaining four years of the Center. Specifically, we have two multifaceted intervention research projects currently in the development phase, one involving modifications to the home environment, and the other involving modifications to individual characteristics, including developing problem‐solving skills, promoting health and well‐being, and building self‐advocacy skills, to support enhanced community participation. Factors identified in the systematic review as most effective in terms of interventions, will be incorporated into these interventions. In addition, our Knowledge Translation project will support continued technical assistance and dissemination of information to consumers wishing to know more about the value of multifaceted interventions. Updating the review will be the responsibility of the Research Director, in cooperation with the Dissemination Coordinator.

## Supporting information

Supporting information
