# Supplementary material for: Multifaceted interventions for supporting community participation among adults with disabilities: A systematic review
Source: Campbell Syst Rev. 2020 Jun 23;16(2):e1092. doi: 10.1002/cl2.1092 (PMC8356358; doi:10.1002/cl2.1092)
Supplement: Supplementary file 1 — Supporting information [file CL2-16-e1092-s001.docx]

**Online supplements**

List of online supplements

National Technical Assistance Center on Transition (NTACT) Quality Indicator Checklists for Group Experimental studies (see http://www.transitionta.org/effectivepractices)

**Other appendices**

**Appendix A. Search Strategies and Terms**

**PubMed search strategies and terms**

Mesh terms were used in PubMed, however, the “explode” command, except for "disabled persons” and “mentally disabled person,” was not utilized. We found the use of the “exact” command to be a more effective search strategy because in tests of searches, the “explode” command resulted in excessive numbers of irrelevant and redundant results.

***PubMed Disability Search*** ***Terms***

| Mentally Disabled Persons[Mesh] |
| --- |
| disabled persons[MesSH] |
| Intellectual Disability[mh:noexp] |
| Vision Disorders[mh:noexp] |
| Communication Disorders[mh:noexp] |
| Visually Impaired Persons [mh:noexp] |
| Amputees[mh:noexp] |
| Mentally Ill Persons [mh:noexp] |
| Persons With Hearing Impairments[mh:noexp] |
| Developmental Disabilities[mh:noexp] |
| Cognition Disorders[mh:noexp] |
| Neurocognitive Disorders[mh:noexp] |
| Mental Disorders[mh:noexp] |
| Mobility Limitation[mh:noexp] |
| Dependent ambulation[mh:noexp] |
| Paraplegia[mh:noexp] |
| quadriplegia[mh:noexp] |
| hearing loss[mh:noexp] |
| blindness[mh:noexp] |
| mental retardation[mh:noexp] |
| Sensory Disorders[mh:noexp] |
| hearing impairment* |
| hearing loss* |
| sensor* disorder* |
| amputee* |
| dependent ambulation* |
| mobility impairment* |
| activity limitation* |
| vision disorder* |
| vision impairment* |
| participation limitation* |
| functional limitation* |
| cognitive impairment* |
| psychiatric disabilit* |
| intellectual disability* |
| mental retard* |

***PubMed Intervention Search Terms***

| Community Integration[Mesh] |
| --- |
| Education, Nonprofessional[Mesh] |
| Rehabilitation[Mesh:NoExp] |
| Recreation Therapy[Majr] |
| Rehabilitation/education[Mesh] |
| lifestyle intervention |
| Psychotherapy[Mesh] |
| Health Promotion[Majr] |
| Mentoring[Majr] |
| Simulation Training[Mesh:NoExp] |

Web of Science Disability Search Terms

| disabled |
| --- |
| developmental near/1 disabilities |
| "communicative disorders" |
| communicative near/1 disorders |
| "mental retardation" |
| Paraplegia |
| Quadriplegia |
| hearing near/1 disorders |
| Deafness |
| hearing near/1 loss |
| vision near/1 disorder |
| Blindness |
| disabled near/2 person |
| disabled near/2 people |
| cognitive near/1 impairment |
| visually near/1 impaired |
| vision near/1 impairment |
| developmental NEAR/1 disabilities |
| mentally disabled person* |
| intellectual near/1 disability |
| mobility near/1 limitation |
| mobility near/1 impairment |
| mental near/1 disorders |
| chronic near/1 "mental disorders" |
| psychiatric near/1 disability |
| mentally near/1 ill |
| mental near/1 health near/1 impairment |
| physical near/1 disability |

***Web of Science Intervention Search Terms (Topic Searches)***

Unlike PubMed and PsycInfo, Web of Science does not have a controlled vocabulary; therefore, the search operator “near” was utilized to establish an accurate, and complete search. Web of Science also requires extensive use of “Web of Science Categories” and “Research Areas” as limiters because of the lack of controlled vocabulary. Two members of the research team manually selected these based on test search results.

|  |
| --- |
| social near/1 support |
| Behavioral near/1 Intervention |
| psychological near/1 intervention |
| training near/1 needs |
| vocational near/1 rehabilitation |
| Vocational and education and training; |
| Home-based near/1 physical near/1 therapy |
| physical near/1 therapy |
| speech near/1 therapy |
| occupational near/1 therapy |
| psychological near/1 rehabilitation |
| rehabilitation near/1 programs |
| community near/1 based near/1 rehabilitation |
| cognitive near/1 behavioral near/1 therapy |
| supported near/1 employment |
| individual near/1 placement near/1 support |
| health near/1 promotion |
| Workshops |
| support near/1 services |
| community near/1 services |
| psychological near/1 therapy |

**PsycInfo search strategies and terms**

In the PsychInfo search we used controlled vocabulary. The term “mentally ill” was not included in the search because the preferred term is “mental disorder.”

***PsycInfo Disability Search Strategy***

| **Search Terms** |
| --- |
| MJSUB.EXACT("Disabilities") |
| su("Developmental Disabilities") |
| MJSUB.EXACT("Multiple Disabilities") |
| MJSUB.EXACT("Physical Disorders") |
| SU("Paraplegia") |
| SU("quadriplegia") |
| [SU.EXACT("special needs")](http://search.proquest.com/recentsearches.recentsearchtabview.recentsearchesgridview.scrolledrecentsearchlist.checkdbssearchlink:rerunsearch/9DED39254B364C29PQ/None?site=psycinfo&t:ac=RecentSearches) |
| MJSUB.EXACT(Mental Disorders) |
| MJSUB.EXACT("Behavior Disorders") |
| psychiatric disabilit* |
| hearing impairment* |
| su(intellectual disabilit*) |
| MJSUB.EXACT("disabled personnel") |
| MJSUB.EXACT("Cognitive Impairment ") |
| subject("Vision Disorders ") |
| subject("Hearing Disorders ") |
| MJSUB.EXACT("Psychiatric Patients ") |
| MJSUB.EXACT("Intellectual Development Disorder") |
| all(quadriplegi*) |
| if(blindness) |
| mjsub(deaf) |
| mjsub(blindness) |
| all(Paraplegi*) |
| if(intellectual disabilit*) |
| if(physical disabilit*) |
| if(mobility impairments) |

***PsycInfo Intervention Search Strategy***

| **Search Term** |
| --- |
| MJSUB.EXACT.EXPLODE("Intervention") |
| SU.EXACT("Treatment") |
| SU.EXACT("Rehabilitation") |
| mjsub(training) |
| if(workshops) |
| SU.EXACT("therapy") |
| if(education) |
| SU.EXACT.EXPLODE("Coaching") |
| if("Coaching") |

**Dissertations & and Theses Abstracts Search Strategy**

Exact("developmental disabilities")

Exact("learning disabilities")

Exact("reading disabilities")

Exact("mental disorders")

Exact("deafness")

Exact("blindness")

Exact("quadriplegia")

Exact("paraplegia")

Exact("behavior disorders")

Exact("special needs")

Exact("hearing impairments")

Exact("intellectual disability")

Exact("hearing disorders")

Exact("vision disorders")

Exact("Cognitive Impairment")

Exact("psychiatric patients")

Exact("mental illness")

all(psychiatric disabilit*)

all(mobility impairment*)

diskw(disabilit*)

or (-)

Exact("intervention")

Exact("rehabilitation")

Exact("employment training programs")

Exact("training")

Exact("therapy")

diskw(coach*)

diskw(workshop*)

diskw(intervent*)

or (-)

and

**Dissertations & and Theses Abstracts Filters:**

Publication Years -present

And

English only

**PolicyFile Search Strategy**

(all(developmental disabilities))

(all(learning disabilities))

(all(reading disabilities))

(all(mental disorders))

(all(deaf*))

(all(blind*))

(all(behavior disorders))

(all(special needs))

(all(hearing impairment))

(all(intellectual disability))

(all(hearing disorder))

(all(vision disorders))

(all(Cognitive Impairment))

(psychiatric patients)

(all(mental illness))

(all(psychiatric disabilit*)

(mobility impairment*)

(all(disabilit*))

or (-)

(intervention)

(rehabilitation)

(employment training programs)

(training)

(therapy)

(coach*)

(workshop*)

(intervent*)

or (-)

and

**PolicyFile Filters**

Publication Years: -Present

And

English only

**Appendix B: Individual Study Outcomes for All Included Studies.**

| **Study** | **Outcome** | **Hedges’ g** | **Variance** |
| --- | --- | --- | --- |
| Bell, 2008 | Competitive hr wk 12 mo follow up | 0.061 | 0.056 |
| Bell, 2008 | Duration of job | 0.109 | 0.056 |
| Bell, 2008 | Number of jobs 12 mo follow up | -0.282 | 0.056 |
| Bell, 2008 | Total hrs wk 12 mo follow up | 0.363 | 0.057 |
| Birk, 2004 | ADL-IADL | -1.078 | 0.187 |
| Birk, 2004 | Depression | -0.850 | 0.185 |
| Birk, 2004 | PANAS NEGATIVE | -1.078 | 0.187 |
| Birk, 2004 | PANAS POSITIVE | 0.221 | 0.182 |
| Birk, 2004 | Perceived Autonomy | 0.850 | 0.185 |
| Cook, 2005 | Achieve employ | 0.185 | 0.003 |
| Cook, 2005 | Earnings | 0.115 | 0.003 |
| Cook, 2005 | Work 40Hr+/month | 0.185 | 0.003 |
| Ferguson, 2012 | Hours per wk | 0.136 | 0.181 |
| Ferguson, 2012 | Wk income | 0.494 | 0.185 |
| Ferguson, 2012 | Ever worked rate | 1.359 | 0.175 |
| Ferguson, 2012 | Monthly wk rate | 1.190 | 0.174 |
| Ferguson, 2012 | Some wk follow Up | 0.812 | 0.171 |
| Fleming, 2009 | MOS Soc Support | 0.241 | 0.070 |
| Fleming, 2009 | SPRS Living | -0.855 | 0.084 |
| Fleming, 2009 | SPRS Occup | -0.781 | 0.084 |
| Fleming, 2009 | SPRS Relation | -0.220 | 0.068 |
| Fleming, 2009 | SPRS Total | -0.755 | 0.079 |
| Gimm, 2011 | Disability Apps | -0.437 | 0.001 |
| Gimm, 2011 | Mean monthly hrs wkd | -0.381 | 0.002 |
| Gimm, 2011 | Not working | 0.139 | 0.002 |
| Guttman, 2009 | Interpersonal Skills | 1.144 | 0.104 |
| Guttman, 2009 | Part Comfort as Student | 1.144 | 0.104 |
| Guttman, 2009 | School Behav Skills | 1.144 | 0.104 |
| Guttman, 2009 | Task Skills | 1.144 | 0.104 |
| Kurz, 2009 | Activities of Daily Living | -0.006 | 0.110 |
| Kurz, 2009 | Mood | 0.677 | 0.121 |
| Kurz, 2009 | Activities of Daily Living | -0.518 | 0.093 |
| Kurz, 2009 | Mood | 0.933 | 0.098 |
| McGurk, 2007 | Number of hours | 0.720 | 0.097 |
| McGurk, 2007 | Number of jobs | 1.413 | 0.114 |
| McGurk, 2007 | Number of weeks | 0.694 | 0.097 |
| McGurk, 2007 | Wages | 0.726 | 0.097 |
| McGurk, 2007 | Total jobs obtained | 0.903 | 0.089 |
| McGurk, 2007 | Worked | 1.049 | 0.089 |
| Mirza, 2009 | Wk performance | 0.565 | 0.055 |
| Onor, 2007 | ADL | 0.483 | 0.165 |
| Onor, 2007 | Depression (GDS) | -1.126 | 0.194 |
| Onor, 2007 | IADL | 0.252 | 0.154 |
| Szanton, 2011 | ADL Difficulty | -0.789 | 0.093 |
| Szanton, 2011 | EQ5D/ health QOL | 0.725 | 0.073 |
| Szanton, 2011 | EuroQOL | 0.955 | 0.080 |
| Szanton, 2011 | IADL Difficulty | -0.587 | 0.075 |
| Tsang, 2009 | Job tenure 11 mo | 1.024 | 0.041 |
| Tsang, 2009 | Job tenure 15 mo | 0.949 | 0.040 |
| Tsang, 2009 | Job tenure 7 mo | 0.957 | 0.040 |
| Tsang, 2009 | Job tenure 11 mo | 0.525 | 0.038 |
| Tsang, 2009 | Job tenure 15 mo | 0.683 | 0.039 |
| Tsang, 2009 | Job tenure 7 mo | 0.486 | 0.038 |
| Tsang, 2009 | Job terminations 11 mo | 1.492 | 0.047 |
| Tsang, 2009 | Job terminations 7 mo | 0.622 | 0.039 |
| Tsang, 2009 | Job terminations15 mo | 0.970 | 0.041 |
| Tsang, 2009 | Salary 11 mo | 0.535 | 0.038 |
| Tsang, 2009 | Salary 15 mo | 0.344 | 0.038 |
| Tsang, 2009 | Salary 7 mo | 0.117 | 0.037 |
| Tsang, 2009 | Job tenure 11 mo | 1.662 | 0.050 |
| Tsang, 2009 | Job tenure 15 mo | 1.798 | 0.053 |
| Tsang, 2009 | Job tenure 7 mo | 1.383 | 0.046 |
| Tsang, 2009 | Employ 11 mo | 0.638 | 0.036 |
| Tsang, 2009 | Employ 15 mo | 0.638 | 0.036 |
| Tsang, 2009 | Employ at 7 mo | 0.572 | 0.036 |
| Tsang, 2009 | Employ 11 mo | 0.606 | 0.037 |
| Tsang, 2009 | Employ 15 mo | 0.647 | 0.037 |
| Tsang, 2009 | Employ at 7 mo | 0.581 | 0.037 |
| Tsang, 2009 | Employ 11 mo | 0.650 | 0.037 |
| Tsang, 2009 | Employ 15 mo | 0.650 | 0.037 |
| Tsang, 2009 | Employ at 7 mo | 0.650 | 0.037 |
| Tsemberis, 2000 | Program | 0.228 | 0.005 |
| Twamley, 2014 | QOL Brief | 0.195 | 0.119 |
| Twamley, 2014 | Working at 14wk | 0.495 | 0.113 |
